# High-flow nasal cannula oxygen versus conventional oxygen therapy for acute respiratory failure due to COVID-19: a systematic review and meta-analysis

**DOI:** 10.1186/s13613-023-01208-8

**Published:** 2023-11-23

**Authors:** Sylvain Le Pape, Sigourney Savart, François Arrivé, Jean-Pierre Frat, Stéphanie Ragot, Rémi Coudroy, Arnaud W. Thille

**Affiliations:** 1 Médecine Intensive Réanimation, Centre Hospitalier Universitaire de Poitiers, 2 rue la Milétrie, 86021 Poitiers Cedex, France; 2 INSERM CIC 1402, IS-ALIVE Research group, University of Poitiers, Poitiers, France

**Keywords:** COVID19, High-flow nasal cannula therapy, Conventional oxygen therapy, Acute hypoxemic respiratory failure, Intubation, Mortality

## Abstract

**Background:**

The effectiveness of high-flow nasal cannula oxygen therapy (HFNC) in patients with acute respiratory failure due to COVID-19 remains uncertain. We aimed at assessing whether HFNC is associated with reduced risk of intubation or mortality in patients with acute respiratory failure due to COVID-19 compared with conventional oxygen therapy (COT).

**Methods:**

In this systematic review and meta-analysis, we searched MEDLINE, Embase, Web of Science, and CENTRAL databases for randomized controlled trials (RCTs) and observational studies comparing HFNC *vs.* COT in patients with acute respiratory failure due to COVID-19, published in English from inception to December 2022. Pediatric studies, studies that compared HFNC with a noninvasive respiratory support other than COT and those in which intubation or mortality were not reported were excluded. Two authors independently screened and selected articles for inclusion, extracted data, and assessed the risk of bias. Fixed-effects or random-effects meta-analysis were performed according to statistical heterogeneity. Primary outcomes were risk of intubation and mortality across RCTs. Effect estimates were calculated as risk ratios and 95% confidence interval (RR; 95% CI). Observational studies were used for sensitivity analyses.

**Results:**

Twenty studies were analyzed, accounting for 8383 patients, including 6 RCTs (2509 patients) and 14 observational studies (5874 patients). By pooling the 6 RCTs, HFNC compared with COT significantly reduced the risk of intubation (RR 0.89, 95% CI 0.80 to 0.98; *p* = 0.02) and reduced length of stay in hospital. HFNC did not significantly reduce the risk of mortality (RR 0.93, 95% CI 0.77 to 1.11;* p* = 0.40).

**Conclusions:**

In patients with acute respiratory failure due to COVID-19, HFNC reduced the need for intubation and shortened length of stay in hospital without significant decreased risk of mortality.

*Trial registration* The study was registered on the International prospective register of systematic reviews (PROSPERO) at https://www.crd.york.ac.uk/prospero/ with the trial registration number CRD42022340035 (06/20/2022).

**Supplementary Information:**

The online version contains supplementary material available at 10.1186/s13613-023-01208-8.

## Introduction

During the COVID-19 pandemic, a surge of patients requiring supplemental oxygen for acute respiratory failure were admitted to hospitals around the world [1–5]. Even though most of these patients were treated with conventional oxygen therapy (COT), several other noninvasive oxygenation strategies have been proposed inside or outside intensive care units (ICUs), including high-flow nasal cannula oxygen therapy (HFNC), continuous positive airway pressure (CPAP), and noninvasive ventilation (NIV) [1, 4, 5].

Exterior to COVID-19, clinical practice guidelines suggest the use of HFNC over COT in patients with acute hypoxemic respiratory failure with the aim of improving comfort and decreasing the risk of intubation [6]. However, level of certainty for this recommendation was low due to small number of clinical trials and conflicting results. Whereas a first randomized controlled trial (RCT) showed a decreased risk of both intubation and death with HFNC as compared to COT in patients with acute respiratory failure mainly due to pneumonia [7], a second large RCT did not find any difference in intubation or mortality in the specific population of immunocompromised patients [8].

In patients with acute respiratory failure due to COVID-19, the first retrospective observational studies conducted in China and then in Europe suggested decreased risk of intubation with HFNC as compared with COT, while no reduction in mortality was observed [9–11]. Although some RCTs confirmed these results, showing lower intubation rates with HFNC than with COT in patients with respiratory failure due to COVID-19 [12, 13], others reported similar intubation rates with both oxygenation supports [14–17]. Another small-scale RCT showed that HFNC reduced both ICU and hospital length of stays as compared with COT [18]. A recent meta-analysis showed decreased risk of intubation with HFNC compared with COT in acute respiratory failure due to COVID-19 [19]. However, only 2 RCTs were included in this meta-analysis and only one of them assessed the risk of intubation, with subsequent high statistical heterogeneity making the meta-analysis conclusions hazardous, as pointed out by its authors.

Therefore, we aimed at assessing the effectiveness of HFNC compared with COT on the risk of intubation and mortality in a large population of patients with acute respiratory failure due to COVID-19. To achieve this objective, we performed a systematic review with meta-analysis of all studies comparing the two oxygenation strategies, including observational studies and RCTs.

## Patients and methods

### Eligibility criteria

We included observational studies and RCTs published in English comparing HFNC (intervention group) with COT (control group) for management of acute respiratory failure due to COVID-19. We excluded pediatric studies, studies that compared HFNC to a noninvasive respiratory support other than COT, and studies that did not mention the risk of intubation and mortality. Records that were not RCTs or observational studies, *i.e.,* protocols, reviews, meta-analyses, opinions, editorials, and case reports were excluded.

### Search strategy

This systematic review with meta-analysis was prospectively registered on PROSPERO (CRD42022340035) and followed the Preferred Reporting Items for a Systematic Reviews and Meta-Analyses (PRISMA) recommendations [20]. Two independent investigators (SLP and SS) conducted an electronic search on databases PubMed/MEDLINE, Embase, Web of Science, the Cochrane Central Register of randomized controlled trials, the Cochrane Central Register of Controlled Trials (CENTRAL) and the Cochrane COVID-19 library for eligible studies from inception to December 1, 2022. The main key search terms were (high-flow oxygen OR high-flow nasal cannula) AND (COVID-19 OR SARS-CoV-2 OR Coronavirus disease-19; Additional file 1: Table S1).

### Study selection

After filtering duplicate records, 2 investigators (SLP and SS) independently screened all identified references for inclusion based on the study title and abstract and reviewed full texts to select the studies. Disagreement during the review process were resolved by discussion or, if necessary, consultation by a third reviewer (AWT).

### Data collection

Data from each included trial, including characteristics of the included studies, design (RCT, prospective observational or retrospective observational), details regarding patients enrolled (demographics and illness severity), details regarding the interventions (fraction of inspired oxygen [FiO_2_] and flow rate), respiratory parameters (respiratory rate, pulsed oxygen saturation [SpO_2_], arterial partial pressure of oxygen [PaO_2_], PaO_2_:FiO_2_ or SpO_2_:FiO_2_ ratio), and outcomes (intubation, length of ICU and hospital stay, time to intubation, and mortality), were independently extracted by 2 investigators (SLP and SS) using a standardized data collection form. There was no imputation for missing data. Variables expressed as median [interquartile range] were converted to mean (standard deviation) as published elsewhere [21]. Discrepancies in the data collection was resolved by discussion or, if necessary, adjudication by a third reviewer (AWT).

### Risk of bias assessment

Risk of bias for each of the included trials was assessed by 2 investigators (SLP and SS) using the Revised Cochrane risk-of-bias tool for randomized trials (RoB2, [22]) and Risk Of Bias In Non-Randomized Studies of Interventions (ROBINS-I, [23]) which considers allocation sequence generation, concealment of allocation, masking of participants and investigators, incomplete outcome reporting, selective outcome reporting, and other sources of bias. Each potential source of bias was graded as high, low, or unclear, which determined whether the studies were considered at high, low, or moderate risk of bias. For evaluation of risk of bias, we focused on RCTs and the two main outcomes (intubation and mortality) and rated the overall risk of bias as the highest risk attributed to any criterion. We assessed the overall certainty of evidence for each outcome using the Grading of Recommendations Assessment, Development and Evaluation (GRADE) framework [24]. Any discrepancy regarding risk of bias and GRADE was solved by discussion and intervention of a third reviewer (AWT) whenever necessary.

## Outcomes

The primary outcome was the need for intubation up to 30 days after randomization. Secondary outcomes included mortality up to 60 days after randomization, length of stay in the ICU and in the hospital, and time to intubation. For sensitivity analyses of non-randomized studies in which the intubation was not reported at day 30 after ICU admission or mortality rate was not reported at day 60 after ICU admission, we collected the intubation or mortality rates at the closest time point and, if absent, we collected the need for intubation and ICU mortality rates.

### Subgroup analyses

For intubation and mortality, two subgroup analyses were performed. In the study type subgroup analysis, outcomes of RCTs were compared to those of prospective and retrospective observational studies. This subgroup analysis was performed as a sensitivity analysis to evaluate whether there were any differences in the results of the main analysis. The second subgroup analysis compared the different outcomes according to the location of patients at inclusion, *i.e.*, ICU or general ward. When possible subgroup effects were suggested, the ICEMAN guidelines were followed to assess their credibility [25].

### Statistical analysis

Binary outcomes were expressed as number of events and continuous data were expressed as mean ± standard deviation. Study weights for binary outcomes were generated using the Mantel–Haenszel fixed-effects model (M-H Fixed) when the *I*^*2*^ statistic was lower than 50% or with the Mantel–Haenszel random-effects model (M-H Random) when the *I*^*2*^ statistic was higher than or equal to 50% [26]. Study weights for continuous outcomes were generated with the inverse variance method with data pooled via fixed-effects (IV Fixed) or random-effects (IV Random) according to the *I*^*2*^ statistic. Subgroup analyses involving observational studies were performed with a random-effects model even if the Higgins *I*^*2*^ statistic was inferior to 50% due to possible unbalanced baseline characteristics. Results were presented as relative risks for binary outcomes and as mean differences for continuous outcomes, both with 95% confidence intervals. Heterogeneity between studies was assessed by the χ^2^ test for homogeneity and the Higgins *I*^*2*^ statistic with a threshold of 50% indicating a substantial heterogeneity for higher values [27]. Small-study effects were assessed by Rücker’s limit meta-analysis method using arcsine difference [28], Peters arcsine test [29]—due to the low heterogeneity variance τ^2^—and visual assessment of the contour-enhanced funnel plots. The certainty in evidence for inconsistency was rated according to the magnitude and direction of heterogeneity. We planned sensitivity analyses for the main outcomes to account for risk of bias of publication. Interaction tests between subgroups were performed to evaluate whether the intervention effect varied among subgroups.

A trial sequential analysis (TSA) was performed to assess the potential for type 1 and type 2 errors caused by scarce data and recurrent testing of accumulated data related to intubation and mortality. A two-sided trial sequential monitoring boundary considering a statistical significance level of 5% and power of 80% with a fixed-effects or random-effects model with the Biggerstaff–Tweedie and DerSimonian–Laird estimators was used. The mean relative risk reduction (RRR) of each outcome was derived from the mean relative risk (RR) of the included studies (15% for both intubation and mortality). The rate of occurrence of each outcome in the control group was computed by pooled incidence analysis of each outcome in the control group of all included studies (50% for intubation and 30% for mortality). Heterogeneity correction based on model variance of the pooled studies was 50% for intubation and 18% for mortality. All analyses were performed in RevMan 5.4 (Cochrane Collaboration, Oxford) software, R software version 4.2.1 [30] using the robvis package for risk-of-bias plots [31], and Trial Sequential Analysis computer TSA V.0.9.5.10 beta.30 [32].

## Results

**Fig. 1:**
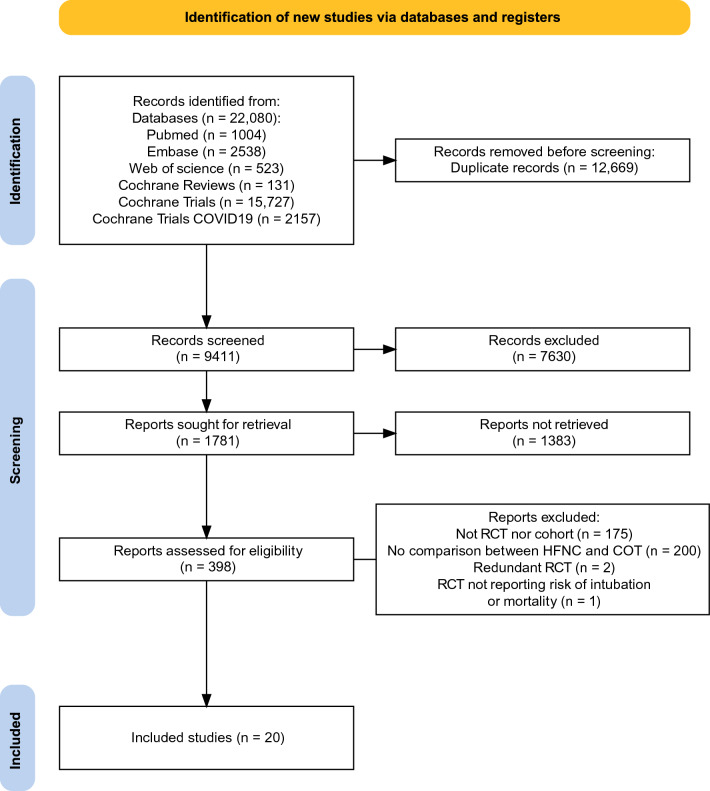
Preferred Reporting Items for Systematic Reviews and Meta-Analyses flow diagram of search strategy and included studies. *COT* conventional oxygen therapy; *HFNC* high-flow nasal cannula therapy; *RCT* randomized controlled trial

We identified a total of 22,080 studies using our search strategy. After removing 12,669 duplicates, 9411 titles were screened, out of which 398 full-text articles were assessed for eligibility. After excluding 377 studies and 1 RCT that did not report the risk of intubation and mortality [18], 20 studies were retained in the analysis for the main and secondary outcomes, including 6 RCTs (2509 patients) [12–17], 5 prospective observational studies (3944 patients) [5, 33–36] and 9 retrospective observational studies (1930 patients) [10, 11, 37–43] (Fig. 1).

### Study characteristics

All RCTs had a clear description of random sequence generation and explained the concealment of allocations. Given the characteristics of the two oxygenation strategies under evaluation, masking of the participants or the attending physicians was not possible. Except for treatment allocation in three studies [14–16], no obvious publication bias was observed among the RCTs in terms of the primary outcome (Additional file 1: Figures S1-S3). Overall bias was moderate to critical for non-randomized studies (Additional file 1: Table S2).

Characteristics of the RCTs and the observational studies included in the analyses are displayed in Table 1 and Additional file 1: Table S3, respectively. Patient characteristics are displayed in Table 2 and Additional file 1: Table S4. Three RCTs included patients exclusively in the ICU, 2 in the ICU and in the wards and 1 in the wards only. Five RCTs had pre-defined intubation criteria (Additional file 1: Table S5), whereas intubation was carried out at the clinician’s judgment in one study [14]. None of the non-randomized studies had pre-defined intubation criteria. Among the 5 RCTs providing PaO_2_:FiO_2_ ratio, 3 RCTs included patients with moderate-to-severe hypoxemia (PaO_2_:FiO_2_ ratio ≤ 200 mm Hg), whereas 2 RCTs also included patients with mild hypoxemia (PaO_2_:FiO_2_ ratio ≤ 300 mm Hg). Mean PaO_2_:FiO_2_ ratio at baseline was 171 ± 67 mm Hg in the HFNC group and 160 ± 49 mm Hg in the COT group, while the mean respiratory rate was 26 ± 3 and 26 ± 3 breaths per min, respectively.

**Table 1:** Study characteristics of the randomized controlled trials

Study, year	Country, N centers, N patients	Enrolment location	Inclusion date	Inclusion criteria	Primary outcomes
Bouadma, 2022	France,19 centers, 224 patients	ICU	April 2020 to January 2021	COVID19 (suspected/confirmed) + AHRF (PaO_2_ < 70 mm Hg or SpO_2_ < 90% in room air, RR > 30/min, labored breathing, respiratory distress, O_2_ > 6L/min)	Time to IMV criteria fulfillment within the first 28 days after randomization
Crimi, 2022	Italy, Greece, Spain, Portugal, Poland, Turkey, 27 centers, 362 patients	Ward	February 2021 to August 2021	COVID19 (confirmed) + Signs of respiratory infection + SpO_2_ ≤ 92% or PaO_2_:FiO_2_ ≤ 300 mm Hg in room air	Rate of escalation of respiratory support to CPAP, NIV or IMV within 28 days of randomization
Frat, 2022	France, 34 centers, 711 patients	ICU	January 2021 to December 2021	COVID19 (suspected/confirmed) + Pulmonary infiltrate + AHRF (PaO_2_:FiO_2_ ≤ 200 mm Hg)	Proportion of patients who died within 28 days following randomization
Nazir, 2022	India, 1 center, 120 patients	ICU	February 2021 to April 2021	COVID19 (confirmed) + AHRF (SpO_2_ ≤ 90% in room air, RR > 30/min) + fever and cough	Progression-free survival without escalation of an oxygen delivery device at day 28
Ospina-Tascón, 2021	Columbia, 3 centers, 199 patients	Ward and ICU	August 2020 to January 2021	COVID19 (suspected/confirmed) + AHRF (PaO2 ≤ 200 mm Hg + use of accessory muscles + RR > 25/min)	Co–primary outcomes were need for intubation and time to clinical recovery within 28 days after randomization
Perkins, 2022	UK, Jersey, 48 centers, 893 patients	Ward and ICU	April 2020 to May 2021	COVID19 (suspected/confirmed) + SpO_2_ ≤ 94% under FiO_2_ ≥ 40%	Composite of tracheal intubation or mortality within 30 days of randomization

*AHRF* acute hypoxemic respiratory failure; *CPAP* continuous positive airway pressure; *COT* conventional oxygen therapy; *CRT* capillary refill time; *CVP* central venous pressure; *FiO*_*2*_ fraction of inspired oxygen; *GCS* Glasgow Coma Scale; *HFNC* high-flow nasal cannula therapy; *ICU* intensive care unit; *IMV* invasive mechanical ventilation; *NIV* noninvasive ventilation; *PaO2* arterial partial pressure of oxygen; *RR* respiratory rate; *SpO*_*2*_ oxygen saturation as measured by pulse oximetry

**Table 2:** Patient characteristics of the randomized controlled trials

Study	Group (N)	Age, y	Sex (male, n, %)	BMI > xx kg/m2	Severity score (admission)	Baseline oxygenation level	Respiratory rate, /min	FiO_2_	Flow rate (L/min)	Proning (n, %)	Cortico-steroids (n, %)	Intubation (n / N, %)	Mortality (n / N, %)	LOS in ICU, d	LOS in hospital, d
Bouadma, 2022	HFNC (115)	65 ± 10	91 (79)	30 ± 5 >30 kg/m2 46 (40%)	SOFA 3 ± 1	SpO_2_ 95 ± 4	24 ± 6	0.79 ± 0.41	50 ± 15	NA	115 (100)	38/115 (33)	47/115 (41)	9.5 ± 7.1	16.6 ± 10.6
	COT (109)	66 ± 9	84 (77)	29 ± 4 > 30 kg/m2 44 (40%)	SOFA 3 ± 1	SpO_2_ 94 ± 3	25 ± 7	NA	15 ± 0	NA	109 (100)	31/109 (28)	41/109 (38)	10.8 ± 11.7	17.6 ± 10.7
Crimi, 2022	HFNC (181)	59 ± 15	119 (66)	29 ± 4 ≥ 30 kg/m2 60 (33%)	NA	PaO_2_:FiO_2_ 271 ± 21	22 ± 3	0.45 ± 0.16	52 ± 9	68 (38)	180 (99)	4/181 (2)	14/181 (8)	10.1 ± 8.5	13.6 ± 8.5
	COT (181)	59 ± 15	112 (62)	28 ± 5 ≥ 30 kg/m2 58 (32%)	NA	PaO_2_:FiO_2_ 276 ± 20	22 ± 4	0.42 ± 0.15 Conversion method not given	NA	70 (39)	180 (99)	7/181 (4)	13/181 (7)	12.1 ± 9.3	16.1 ± 15.1
Frat, 2022	HFNC (357)	61 ± 12	250 (70)	29 ± 5	SAPS II 31 ± 10	PaO_2_:FiO_2_ 128 ± 31	28 ± 6	0.58 ± 0.08	51 ± 10	98 (28)	338 (95)	160/357 (45)	36/357 (10)	12.8 ± 14.0	18.4 ± 11.5
	COT (354)	61 ± 12	247 (70)	29 ± 6	SAPS II 29 ± 13	PaO_2_:FiO_2_ 132 ± 31	29 ± 6	0.58 ± 0.07	13 ± 3	83 (24)	335 (95)	186/354 (52)	40/354 (11)	13.9 ± 15.5	19.7 ± 14.3
Nazir, 2022	HFNC (60)	54 ± 12	28 (47)	NA	NA	PaO_2_:FiO_2_ 207 ± 5	28 ± 1	NA	NA	48 (80)	56 (93)	2/60 (3)	3/60 (5)	NA	NA
	COT (60)	57 ± 3	32 (53)	NA	NA	PaO_2_:FiO_2_ 208 ± 4	28 ± 1	NA	NA	45 (75)	58 (97)	8/60 (13)	5/60 (8)	NA	NA
Ospina-Tascón, 2021	HFNC (99)	59 ± 14	71 (72)	29 ± 4	SOFA 10 ± 3	PaO_2_:FiO_2_ 112 ± 38	29 ± 4	0.63 ± 0.09	60 ± 0	74 (75)	4 (4)	34/99 (34)	8/99 (8)	10.2 ± 8.5	15.8 ± 10.5
	COT (100)	57 ± 13	63 (63)	30 ± 5	SOFA 10 ± 3	PaO_2_:FiO_2_ 117 ± 47	29 ± 4	0.85 ± 0.00	15 ± 0	64 (64)	10 (10)	51/100 (51)	16/100 (16)	15.3 ± 18.6	18.7 ± 15.1
Perkins, 2022	HFNC (418)	58 ± 13	272 (65)	> 35 kg/m2 81 (19%)	NA	PaO_2_:FiO_2_ 136 ± 81	25 ± 7	0.60 ± 0.26	52 ± 2	243/341 (71%)	NA	170/415 (41)	78/416 (19)	10.5 ± 15.6	18.3 ± 20.0
	COT (475)	58 ± 13	312 (66)	> 35 kg/m2 75 (16%)	NA	PaO_2_:FiO_2_ 124 ± 53	24 ± 6	0.60 ± 0.26	NA	252/374 (67%)	NA	153/368 (42)	74/370 (20)	9.6 ± 14.1	17.1 ± 18.0

Categorical data are expressed as number of patients (percentage of total group size). Continuous data are expressed as mean ± standard deviation

*BMI* body mass index; *COT* conventional oxygen therapy; *FiO*_*2*_ fraction of inspired oxygen; *HFNC* high-flow nasal cannula therapy; *NA* not available data; *PaO*_*2*_ arterial partial pressure of oxygen; *SpO*_*2*_ oxygen saturation as measured by pulse oximetry

### Primary outcome: risk of intubation

**Fig. 2:**
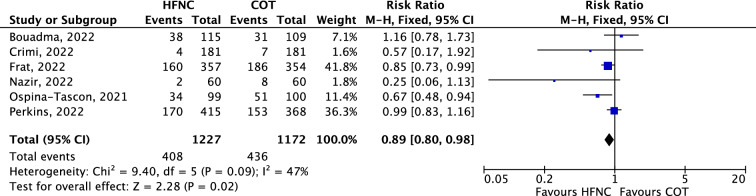
Forest plot of intubation rate comparison between HFNC and COT from randomized controlled trials (fixed-effects meta-analysis by the Mantel–Haenszel method). *COT* conventional oxygen therapy; *HFNC* high-flow nasal cannula; *M-H* Mantel–Haenszel

Of the 6 RCTs analyzed for the primary outcome, the pooled estimates showed that HFNC significantly reduced the need for intubation compared with COT (RR 0.89, 95% CI 0.80 to 0.98; *p* = 0.02; Fig. 2). The sensitivity analysis made on the 10 observational studies in which intubation rate was reported also revealed a reduced risk of intubation with HFNC as compared with COT (RR 0.79, 95% CI 0.73 to 0.86; p < 0.001; Additional file 1: Figure S4). A sensitivity analysis through a leave-one-out approach including all studies (RCTs and observational studies) showed consistent reduction in the risk of intubation in the HFNC group (Additional file 1: Figures S5 and S6). Subgroup analyses showed no significant differences in the risks of intubation between patients treated in the ICU (RR 0.89, 95% CI 0.61 to 1.30; *p* = 0.55) and those treated in the general wards (RR 0.82, 95% CI 0.59 to 1.14; *p* = 0.24; Additional file 1: Figure S7).

**Fig. 3:**
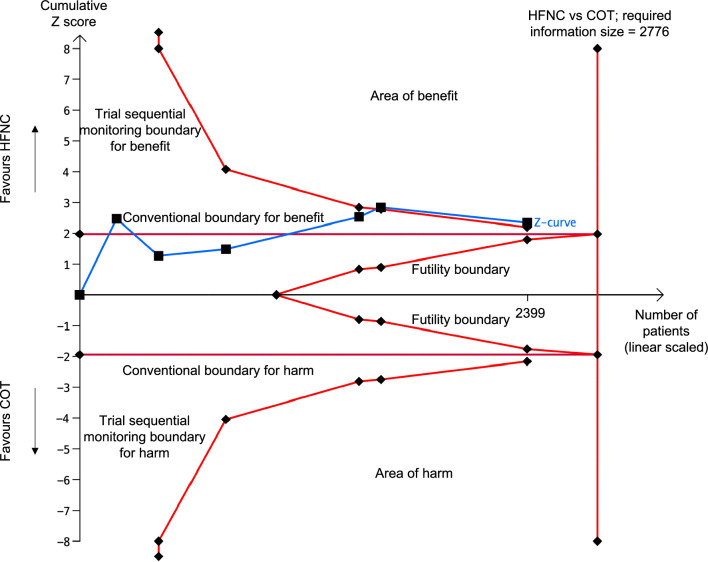
Trial sequential analysis of intubation outcome. Studies are shown as black-filled squares on the cumulative Z curve. For the conventional boundaries, *p* = 0.05 and z =|1.96|. The TSA software only generates Z scores from − 8 to + 8. The cumulative Z curve crosses the conventional boundary for benefit and the trial sequential monitoring boundary for benefit without reaching the required information size line at n = 2776 showing that, compared with COT, HFNC has clinical benefit, leading to reduced risk of intubation. *COT* conventional oxygen therapy; *HFNC* high-flow nasal cannula; *TSA* trial sequential analysis

**Fig. 4:**
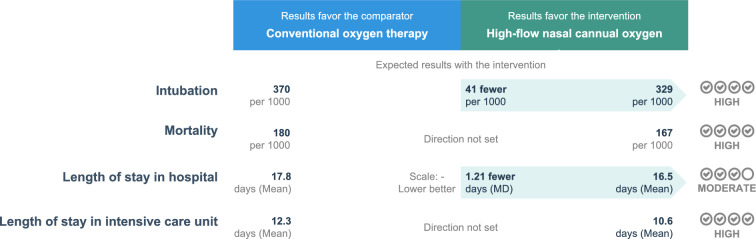
GRADE evidence profile for the studies in the meta-analysis

In the trial sequential analysis, the cumulative Z-curve exceeded the boundary for benefit before reaching required information size suggesting a definitive conclusion (Fig. 3). The quality of evidence on intubation was high with low heterogeneity and no serious inconsistency (Fig. 4). The GRADE assessment for the certainty of evidence is summarized in Additional file 1: Table S6.

### Secondary outcomes

**Fig. 5:**
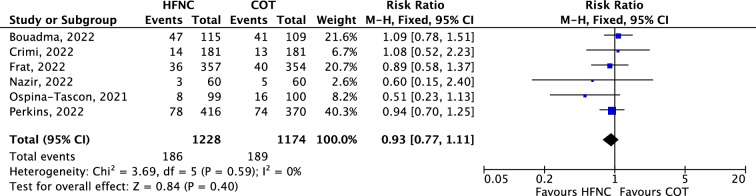
Forest plot of mortality comparison between HFNC and COT from randomized controlled trials (fixed-effects meta-analysis by the Mantel–Haenszel method). *COT* conventional oxygen therapy; *HFNC* high-flow nasal cannula; *M-H* Mantel–Haenszel

HFNC was not associated with reduced risk of mortality as compared with COT in the 6 RCTs (RR 0.93, 95% CI 0.77 to 1.11;* p* = 0.40; Fig. 5), nor in the 5 prospective observational studies (RR 1.16, 95% CI 0.92 to 1.46; *p* = 0.21), whereas it was associated with decreased risk of mortality in the 9 retrospective observational studies (RR 0.83, 95% CI 0.71 to 0.98; *p* = 0.03; Additional file 1: Figure S8). The mortality of patients treated with HFNC or COT did not differ in ICUs (RR 0.97, 95% CI 0.75 to 1.26; *p* = 0.81) or in the general wards (RR 0.89 95% CI 0.69 to 1.14; *p* = 0.36; Additional file 1: Figure S9).

In the trial sequential analysis, the futility boundaries were reached, suggesting that HFNC is unlikely to have an effect on mortality and that the addition of more trials would not modify the conclusion (Additional file 1: Figure S10).

Among the 5 RCTs in which it was reported, HFNC was not associated with reduced length of stay in ICU (mean difference − 1.34 day, 95% CI − 2.86 to 0.19; *p* = 0.09) but reduced length of stay in hospital (mean difference − 1.21 day, 95% CI − 2.34 to − 0.07; *p* = 0.04) as compared with COT (Additional file 1: Figures S11 and S12).

Three RCTs [12–14] reported the time from randomization to intubation and none of them showed a significant difference between groups (mean difference 0.23 days, 95% CI − 0.25 to 0.71; *p* = 0.34).

In terms of safety, no serious adverse event was reported in the HFNC group [12–15].

## Discussion

In this systematic review and meta-analysis including patients with acute respiratory failure due to COVID-19, HFNC significantly decreased the risk of intubation with a high certainty of evidence, without significantly decreased risk of death as compared with COT.

### Effect of HFNC on intubation and quality of evidence

By pooling the 6 RCTs detailing intubation rates, we found a decreased risk of intubation with HFNC compared with COT. While two RCTs showed a decreased risk of intubation with HFNC rather than COT, the 4 other RCTs did not show any significant difference.

From the start of the pandemic, several observational studies have reported a decreased risk of intubation with HFNC rather than COT first in China, and then in Europe [9–11]. After which, 2 large-scale RCTs including patients mainly admitted to ICUs showed lower intubation rates with HFNC rather than COT [12, 13]. These 2 clinical trials included patients with severe respiratory failure, *i.e.*, patients with moderate-to-severe hypoxemia (PaO_2_: FiO_2_ ≤ 200 mm Hg), respiratory rate greater than 25 breaths/minute or activation of accessory respiratory muscles. Interestingly, the intubation rates observed in these trials (30 to 50%) were very close to those reported in the FLORALI trial, which included non-COVID-19 patients with similar respiratory severity [7]. However, two other large-scale clinical trials did not show any difference regarding intubation rates between HFNC and COT [14, 15]. Nevertheless, one limitation of these two trials is that around one-fourth of the patients randomized in the control group received HFNC or CPAP in the place of COT, which may have mitigated the potential benefits of HFNC. Moreover, the study by Perkins *et al.* was conducted in the UK from April 2020: at that time, the peak of the epidemic had been reached, ICUs were overwhelmed, and only 60% of the patients included were admitted to ICUs whereas the others were treated in general wards. Another RCT included 362 patients treated in general wards where 14% of the patients randomized in the control group received HFNC and no significant difference regarding intubation rates was found in patients treated with HFNC or COT [16]. Lastly, one RCT was underpowered to detect a difference in intubation rate even though it was limited to patients admitted in ICUs [17].

The surge of patients with acute respiratory failure during the pandemic led intensivists to treat a large number of patients with noninvasive oxygenation strategies outside ICUs due to the limited number of available ICU beds [44]. Management of patients with acute respiratory failure outside ICUs may have had an impact on the effect of oxygenation strategies as decreased monitoring in a general ward may influence the decision for intubation. In keeping with this, in the Perkins trial the risk of intubation or mortality was significantly lower with HFNC than with COT in the subgroup of patients receiving higher FiO_2_ (above 60%), *i.e.*, in patients with greater respiratory disease severity [14]. However, we did not find a difference in risk of intubation with HFNC compared with COT between trials conducted in ICUs and general wards. This suggests that in the pandemic context, the surge of patients requiring intensive oxygen supports could be treated outside of ICUs, but this requires confirmation by further dedicated large-scale RCTs.

A first meta-analysis suggested that HFNC may reduce intubation rate and 28-day ICU mortality compared with COT [19]. However, due to high statistical heterogeneity (*I*^*2*^ of 85% for the meta-analysis of the risk of intubation), the quality of evidence was low and the trial sequential analysis of risk of intubation relied mainly on non-randomized studies. Out of the 3370 included patients, only 221 were from RCTs. This deeply weakened the conclusions as pointed out by the authors, who indicated that large-scale randomized controlled trials were necessary to validate their findings. To the best of our knowledge, our meta-analysis is the first to explore all available data on COVID-19-related acute hypoxemic respiratory failure comparing the effect of HFNC to that of COT by pooling 6 RCTs for the main outcomes and pooling 5 prospective studies and 9 retrospective studies for sensitivity analyses. According to the GRADE methodology, HFNC was associated with decreased risk of intubation as compared with COT with high evidence.

### Effect of HFNC on mortality and in-ICU or in-hospital length of stay

No RCT showed improved survival with HFNC or any other noninvasive oxygenation strategy as compared with COT in patients with acute respiratory failure due to COVID-19 [12–17, 45–47]. By pooling all RCTs in our meta-analysis, we confirmed the absence of effect of HFNC on mortality. Our findings on risks of intubation and mortality are in line with the meta-analysis by Rochwerg *et al.*, who which also found a lower risk of intubation in patients treated with HFNC than in those treated with COT, without impacting mortality in the context of acute hypoxemic respiratory failure not related to COVID-19, thereby reinforcing the external validity of our findings [48].

Before the COVID-19 pandemic, clinical practice guidelines suggested the use of HFNC rather than COT in patients with acute respiratory failure [6]. These recommendations were driven mainly by the FLORALI trial, which was the seminal study showing that as compared with COT, HFNC reduced mortality in patients with acute hypoxemic respiratory failure [7]. This study also reported a reduced intubation rate in patients treated with HFNC as compared with those treated with COT, but only in those with moderate-to-severe hypoxemia (PaO_2_:FiO_2_ ≤ 200 mm Hg) [7].

Finally, we found that compared with COT, HFNC was associated with reduced in-hospital length of stay without changing in-ICU length of stay. Length of stay in hospital is not only important at the individual level, but is also relevant at a community healthcare level, given the fact that hospitals were overloaded during the pandemic.

## Limitations

This study has several limitations. First, a general assumption of meta-analysis is that both the enrolled population and the intervention protocols of each individual trial were similar across different studies. However, the flow rate in the COT group was not specified in 3 RCTs, and FiO_2_ under HFNC was not available in 1 RCT. Nonetheless, our findings were consistent in sensitivity analyses that excluded these trials with a leave-one-out strategy. As expected, the meta-analysis was sensitive to the two studies—Ospina-Tascón *et al.* [12] and Frat *et al.* [13]—that found a statistically significant difference in intubation rate between HFNC and COT groups. Second, the nature of the evaluated treatments prevents blinding the participants or the treating clinicians. While the assessment of mortality is likely to be unbiased, the clinical judgment of intubation criteria may have differed according to the oxygenation strategies implemented. Nonetheless, except for one RCT, each study described pre-defined intubation criteria, thereby reducing this risk of bias. Third, a large-scale RCT reported intubation as cumulative incidence of outcome [15]. To overcome this limitation, we attempted to contact the main authors by email to obtain the mortality and intubation rates at day 28 but did not receive any replies. For that reason, in our analyses we computed these cumulative incidences as rates. Although the rates and cumulative incidences are often similar, uncertainty may result. However, based on the sensitivity analysis, exclusion of this trial did not change our findings.

The absence of effect of HFNC on mortality may be explained by several factors. First, the rate of crossover from COT to HFNC or to another noninvasive respiratory support in the control group was not uncommon (between 15 and 30% of cases in 3 RCTs [14–16]), which may have reduced the observed effect size of an effective treatment. Second, the use of steroids or other immunomodulatory drugs was highly variable from one study to another and may have mitigated the differences. Third, we cannot rule out the possibility that mortality of patients requiring intubation while receiving a noninvasive respiratory support could be higher than in those intubated while receiving only COT. Lastly, whereas awake prone position may reduce the risk of intubation in patients with acute respiratory failure due to COVID-19 [49], the proportion of patients who were prone did not significantly differ between those treated with HFNC and those treated with COT, making unlikely the impact of awake prone positioning on the absence of mortality found in our study.

## Conclusion

In this systematic review and meta-analysis pooling studies including patients with acute respiratory failure due to COVID-19, HFNC was associated with lower risk of intubation and reduced length of stay in hospital without any effect on mortality as compared with COT. Our findings support the routine implementation of HFNC in patients admitted to the ICUs for acute respiratory failure due to COVID-19.

### Supplementary Information

**Additional file 1: Figure S1.** Risk of bias graph (ROB 2) for intubation outcome from randomized controlled trials. **Figure S2.** Funnel plot for intubation rate and assessment of small-study effects by Rücker’s limit meta-analysis method using Arcsine difference and Peters arcsine test. **Figure S3.** Funnel plot for mortality rate and assessment of small-study effects by Rücker’s limit meta-analysis method using arcsine difference and Peters arcsine test. **Figure S4.** Forest plot of intubation rate comparison between HFNC and COT from prospective and retrospective studies (random-effects meta-analysis by the Mantel–Haenszel method). COT, conventional oxygen therapy; HFNC, high-flow nasal cannula; M-H, Mantel–Haenszel. **Figure S5.** Sensitivity analysis of the risk of intubation through the leave-one-out strategy for the randomized controlled trials (fixed-effects meta-analysis by the Mantel–Haenszel method). COT, conventional oxygen therapy; HFNC, high-flow nasal cannula. **Figure S6.** Sensitivity analysis of the risk of intubation through the leave-one-out strategy for all studies (random-effects meta-analysis by the Mantel–Haenszel method). COT, conventional oxygen therapy; HFNC, high-flow nasal cannula. **Figure S7.** Forest plot of intubation rate comparison between HFNC and COT from randomized controlled trials according to the location of admission (random-effects meta-analysis by the Mantel–Haenszel method). COT, conventional oxygen therapy; HFNC, high-flow nasal cannula; ICU, intensive care unit; M-H, Mantel–Haenszel. **Figure S8.** Forest plot of mortality comparison between HFNC and COT from prospective and retrospective studies (random-effects meta-analysis by the Mantel–Haenszel method). COT, conventional oxygen therapy; HFNC, high-flow nasal cannula; M-H, Mantel–Haenszel. **Figure S9.** Forest plot of mortality rate comparison between HFNC and COT from randomized controlled trials according to the location of admission (fixed-effects meta-analysis by the Mantel–Haenszel method). COT, conventional oxygen therapy; ICU, intensive care unit; HFNC, high-flow nasal cannula; M-H, Mantel–Hanszel. **Figure S10**. Trial sequential analysis of mortality outcome. Studies are shown as black-filled squares on the cumulative Z curve. For the conventional boundaries, *p*=0.05 and z=|1.96|. The TSA software only generates Z scores from -8 to +8. The cumulative Z curve crosses the futility boundary, suggesting that HFNC is unlikely to have an effect on mortality in comparison to COT and that the addition of more trials would not modify the conclusion. COT, conventional oxygen therapy; HFNC, high-flow nasal cannula; TSA, trial sequential analysis. **Figure S11.** Forest plot of intensive care unit length of stay comparison between HFNC and COT from randomized controlled trials (random-effects meta-analysis by the inverse variance method). COT, conventional oxygen therapy; HFNC, high-flow nasal cannula; IV, inverse variance. **Figure S12**. Forest plot of hospital length of stay comparison between HFNC and COT from randomized controlled trials (fixed-effects meta-analysis by the inverse variance method). COT, conventional oxygen therapy; HFNC, high-flow nasal cannula; IV, inverse variance. **Table S1.** Search strategy. **Table S2.** Risk of bias graph (ROBINS-I) for intubation outcome from non-randomized controlled trials. **Table S3.** Study characteristics of the non-randomized controlled trials. **Table S4.** Patient characteristics of the non-randomized controlled trials. **Table S5.** Pre-defined intubation criteria of the randomized controlled trials. **Table S6.** GRADE evidence profile for the studies in the meta-analysis.

## Data Availability

Data are available from the corresponding author on reasonable request.
