# Supplementary material for: High-flow nasal cannula oxygen versus conventional oxygen therapy for acute respiratory failure due to COVID-19: a systematic review and meta-analysis
Source: Ann Intensive Care. 2023 Nov 23;13:114. doi: 10.1186/s13613-023-01208-8 (PMC10667189; doi:10.1186/s13613-023-01208-8)
Supplement: Supplementary file 1 — Additional file 1: Figure S1. Risk of bias graph (ROB 2) for intubation outcome from randomized controlled trials. Figure S2. Funnel plot for intubation rate and assessment of small-study effects by Rücker’s limit meta-analysis method using Arcsine difference and Peters arcsine test. Figure S3. Funnel plot for mortality rate and assessment of small-study effects by Rücker’s limit meta-analysis method using arcsine difference and Peters arcsine test. Figure S4. Forest plot of intubation rate comparison between HFNC and COT from prospective and retrospective studies (random-effects meta-analysis by the Mantel–Haenszel method). COT, conventional oxygen therapy; HFNC, high-flow nasal cannula; M-H, Mantel–Haenszel. Figure S5. Sensitivity analysis of the risk of intubation through the leave-one-out strategy for the randomized controlled trials (fixed-effects meta-analysis by the Mantel–Haenszel method). COT, conventional oxygen therapy; HFNC, high-flow nasal cannula. Figure S6. Sensitivity analysis of the risk of intubation through the leave-one-out strategy for all studies (random-effects meta-analysis by the Mantel–Haenszel method). COT, conventional oxygen therapy; HFNC, high-flow nasal cannula. Figure S7. Forest plot of intubation rate comparison between HFNC and COT from randomized controlled trials according to the location of admission (random-effects meta-analysis by the Mantel–Haenszel method). COT, conventional oxygen therapy; HFNC, high-flow nasal cannula; ICU, intensive care unit; M-H, Mantel–Haenszel. Figure S8. Forest plot of mortality comparison between HFNC and COT from prospective and retrospective studies (random-effects meta-analysis by the Mantel–Haenszel method). COT, conventional oxygen therapy; HFNC, high-flow nasal cannula; M-H, Mantel–Haenszel. Figure S9. Forest plot of mortality rate comparison between HFNC and COT from randomized controlled trials according to the location of admission (fixed-effects meta-analysis by the Mantel–Haenszel method). [file 13613_2023_1208_MOESM1_ESM.zip › Supplementary/Supplementary table S1.docx]

**Supplementary table S1. Search strategy.**

**Cochrane Trials and Cochrane Reviews:**

#1 high flow oxygen:ti,ab,kw OR high flow nasal cannula therapy:ti,ab,kw AND coronavirus disease 2019:ti,ab,kw OR covid-19:ti,ab,kw OR SARS-CoV-2:ti,ab,kw

**Cochrane COVID19:**

#1 high and flow and oxygen

**Embase:**

#1 ('high flow oxygen' OR 'high flow nasal cannula therapy') AND ('covid19' OR 'coronavirus disease 2019' OR 'sars-cov-2') AND [01-01-2019]/sd NOT [02-11-2022]/sd

**Pubmed:**

#1 (((high flow nasal cannula therapy) OR (high flow oxygen)) AND ((covid19) OR (coronavirus disease 2019) OR (SARS-CoV-2))) AND (("2019/01/01[Date - Publication] : "2022/11/01"[Date - Publication]))

**Web of science:**

#1 ((((ALL=(high flow nasal cannula therapy)) OR ALL=(high flow oxygen)) AND (ALL=(coronavirus disease 2019) OR ALL=(covid19) OR ALL=(SARS-CoV-2))) AND DOP=(2019-01-01/2022-11-01)
