# Supplementary material for: High-flow nasal cannula oxygen versus conventional oxygen therapy for acute respiratory failure due to COVID-19: a systematic review and meta-analysis
Source: Ann Intensive Care. 2023 Nov 23;13:114. doi: 10.1186/s13613-023-01208-8 (PMC10667189; doi:10.1186/s13613-023-01208-8)
Supplement: Supplementary file 1 — Additional file 1: Figure S1. Risk of bias graph (ROB 2) for intubation outcome from randomized controlled trials. Figure S2. Funnel plot for intubation rate and assessment of small-study effects by Rücker’s limit meta-analysis method using Arcsine difference and Peters arcsine test. Figure S3. Funnel plot for mortality rate and assessment of small-study effects by Rücker’s limit meta-analysis method using arcsine difference and Peters arcsine test. Figure S4. Forest plot of intubation rate comparison between HFNC and COT from prospective and retrospective studies (random-effects meta-analysis by the Mantel–Haenszel method). COT, conventional oxygen therapy; HFNC, high-flow nasal cannula; M-H, Mantel–Haenszel. Figure S5. Sensitivity analysis of the risk of intubation through the leave-one-out strategy for the randomized controlled trials (fixed-effects meta-analysis by the Mantel–Haenszel method). COT, conventional oxygen therapy; HFNC, high-flow nasal cannula. Figure S6. Sensitivity analysis of the risk of intubation through the leave-one-out strategy for all studies (random-effects meta-analysis by the Mantel–Haenszel method). COT, conventional oxygen therapy; HFNC, high-flow nasal cannula. Figure S7. Forest plot of intubation rate comparison between HFNC and COT from randomized controlled trials according to the location of admission (random-effects meta-analysis by the Mantel–Haenszel method). COT, conventional oxygen therapy; HFNC, high-flow nasal cannula; ICU, intensive care unit; M-H, Mantel–Haenszel. Figure S8. Forest plot of mortality comparison between HFNC and COT from prospective and retrospective studies (random-effects meta-analysis by the Mantel–Haenszel method). COT, conventional oxygen therapy; HFNC, high-flow nasal cannula; M-H, Mantel–Haenszel. Figure S9. Forest plot of mortality rate comparison between HFNC and COT from randomized controlled trials according to the location of admission (fixed-effects meta-analysis by the Mantel–Haenszel method). [file 13613_2023_1208_MOESM1_ESM.zip › Supplementary/Supplementary table S2.docx]

**Supplementary table S2. Risk of bias graph (ROBINS-I) for intubation outcome from non-randomized controlled trials.**

| **Study** | **Bias due to confounding** | **Bias in selection of participants into the study** | **Bias in classification of interventions** | **Bias due to deviations from intended interventions** | **Bias due to missing data** | **Bias in measurement of outcomes** | **Bias in selection of the reported result** | **Overall Bias** |
| --- | --- | --- | --- | --- | --- | --- | --- | --- |
| **COVID-ICU group, 2021** | Low | Moderate | Low | Low | Moderate | Low | Moderate | Moderate |
| **Roger, 2021** | Low | Moderate | Low | Low | Low | Low | Low | Moderate |
| **Wendel-Garcia, 2021** | Low | Moderate | Low | Low | Low | Low | Moderate | Moderate |
| **ACCCOS, 2021** | Low | Moderate | Critical | Low | Low | Low | Low | Critical |
| **Hansen, 2021** | Low | Moderate | Low | Low | Low | Low | Moderate | Moderate |
| **Bonnet, 2021** | Low | Moderate | Moderate | Low | Low | Low | Moderate | Moderate |
| **Demoule 2020** | Low | Moderate | Moderate | Low | Low | Low | Moderate | Moderate |
| **Gallardo, 2022** | Low | Moderate | Moderate | Low | Low | Low | Moderate | Moderate |
| **Hacquin, 2021** | Low | Moderate | Moderate | Low | Low | Low | Moderate | Moderate |
| **Kabak, 2021** | Serious | Moderate | Moderate | Low | Low | Low | Moderate | Serious |
| **Kamil, 2023** | Low | Moderate | Moderate | Low | Low | Low | Moderate | Moderate |
| **Liao, 2020** | Serious | Moderate | Moderate | Low | Low | Critical | Low | Critical |
| **Sayan, 2021** | Serious | Moderate | Moderate | Low | Low | Low | Moderate | Serious |
| **Wendel‑Garcia, 2022** | Low | Moderate | Moderate | Low | Low | Low | Moderate | Moderate |
