# Supplementary material for: High-flow nasal cannula oxygen versus conventional oxygen therapy for acute respiratory failure due to COVID-19: a systematic review and meta-analysis
Source: Ann Intensive Care. 2023 Nov 23;13:114. doi: 10.1186/s13613-023-01208-8 (PMC10667189; doi:10.1186/s13613-023-01208-8)
Supplement: Supplementary file 1 — Additional file 1: Figure S1. Risk of bias graph (ROB 2) for intubation outcome from randomized controlled trials. Figure S2. Funnel plot for intubation rate and assessment of small-study effects by Rücker’s limit meta-analysis method using Arcsine difference and Peters arcsine test. Figure S3. Funnel plot for mortality rate and assessment of small-study effects by Rücker’s limit meta-analysis method using arcsine difference and Peters arcsine test. Figure S4. Forest plot of intubation rate comparison between HFNC and COT from prospective and retrospective studies (random-effects meta-analysis by the Mantel–Haenszel method). COT, conventional oxygen therapy; HFNC, high-flow nasal cannula; M-H, Mantel–Haenszel. Figure S5. Sensitivity analysis of the risk of intubation through the leave-one-out strategy for the randomized controlled trials (fixed-effects meta-analysis by the Mantel–Haenszel method). COT, conventional oxygen therapy; HFNC, high-flow nasal cannula. Figure S6. Sensitivity analysis of the risk of intubation through the leave-one-out strategy for all studies (random-effects meta-analysis by the Mantel–Haenszel method). COT, conventional oxygen therapy; HFNC, high-flow nasal cannula. Figure S7. Forest plot of intubation rate comparison between HFNC and COT from randomized controlled trials according to the location of admission (random-effects meta-analysis by the Mantel–Haenszel method). COT, conventional oxygen therapy; HFNC, high-flow nasal cannula; ICU, intensive care unit; M-H, Mantel–Haenszel. Figure S8. Forest plot of mortality comparison between HFNC and COT from prospective and retrospective studies (random-effects meta-analysis by the Mantel–Haenszel method). COT, conventional oxygen therapy; HFNC, high-flow nasal cannula; M-H, Mantel–Haenszel. Figure S9. Forest plot of mortality rate comparison between HFNC and COT from randomized controlled trials according to the location of admission (fixed-effects meta-analysis by the Mantel–Haenszel method). [file 13613_2023_1208_MOESM1_ESM.zip › Supplementary/Supplementary table S3.docx]

**Supplementary table S3. Study characteristics of the non-randomized controlled trials.**

1. **Prospective observational studies**

| **Study name** | **Country,**  **N centers,**  **N patients** | **Enrolment location** | **Inclusion date** | **Inclusion criteria** | **Outcomes** | **Intubation criteria** |
| --- | --- | --- | --- | --- | --- | --- |
| **COVID-ICU group, 2021** | France, Belgium, and Switzerland  **137 centers**  **1333 patients** | ICU | February to May 2020 | COVID19 (confirmed) | Patient outcomes included the date of invasive mechanical ventilation, the date of ICU and hospital discharge, and vital status at ICU discharge, hospital discharge, and 28, 60, and 90 days after ICU admission. | NA |
| **Roger, 2021** | France  **29 centers**  **407 patients** | ICU | March to July 2020 | COVID19 (suspected/confirmed) | Detailed description of the initial management of COVID-19 patients admitted to French ICUs.  Risk factors of 28-day mortality | NA |
| **Wendel-Garcia, 2021** | Andorra, Austria, Ecuador, France, Germany, Hungary, Italy, Neatherlands, Spain, Switzerland  **63 centers**  **172 patients** | ICU | March to September 2020 | COVID19 (suspected/confirmed)  +  AHRF (COT ≥10 L/min, HFNC, NIV, or IMV at the time point of admission to the ICU defined as day 0). | Requirement of intubation, Withdrawal of life supporting therapies, ICU length of stay, days, ICU mortality | NA |
| **ACCCOS, 2021** | Egypt, Ethiopia, Ghana, Kenya, Libya, Malawi, Mozambique, Niger, Nigeria and South Africa  **64 centers**  **1941 patients** | ICU | May to December 2020 | COVID19 (suspected/confirmed) | In-hospital mortality censored at 30 days. factors (ie, human and facility resources, patient comorbidities, and critical care interventions) that were associated with mortality in these adult patients. | NA |
| **Hansen, 2021** | USA  **1 center**  **91 patients** | ICU | March to May 2020 | COVID19 (confirmed)  +  AHRF requiring invasive mechanical ventilation | Mortality at day 28, ICU length of stay, Ventilator free days | Mechanical ventilation was initiated at the discretion of the treating providers. |

AHRF: acute hypoxemic respiratory failure; COT: conventional oxygen therapy; FiO_2_: fraction of inspired oxygen; HFNC: high-flow nasal cannula therapy; ICU: intensive care unit; IMV: invasive mechanical ventilation; NIV: noninvasive ventilation; PaO_2_: arterial partial pressure of oxygen; SpO_2_: oxygen saturation as measured by pulse oximetry

1. **Retrospective observational studies**

| **Study** | **Country,**  **Participating centers (C),**  **Patients (P)** | **Enrolment location** | **Inclusion date** | **Inclusion criteria** | **Primary outcomes** | **Intubation**  **criteria** |
| --- | --- | --- | --- | --- | --- | --- |
| **Bonnet, 2021** | France  **2 centers**  **138 patients** | ICU | March to May 2020 | COVID19 (suspected/confirmed)  +  AHRF (respiratory rate of more than 25 per minute and need for standard oxygen ≥ 3L/min to maintain SpO_2_ ≥ 92%) | The primary outcome was the rate of IMV after ICU admission. Secondary outcomes were death at day 28 and day 60, length of ICU stay and ventilator-free days at day 28. | NA |
| **Demoule 2020** | France  **4 centers**  **379 patients** | ICU | February to April 2020 | COVID19 (confirmed)  +  AHRF (respiratory rate >25/min, bilateral pulmonary infiltrates on chest X-ray or computed tomography scan, and need for standard oxygen >3 L/min to maintain peripheral arterial oxygen saturation >92%) | Invasive mechanical ventilation at Day 28, ICU mortality, Mortality at Day 28, Mortality at Day 60 | NA |
| **Gallardo, 2022** | Argentina  **1 center**  **84 patients** | NA | NA | COVID19 (confirmed)  +  AHRF (not defined) | Intubation rate  Time from hospital admission to intubation (days)  Duration of invasive mechanical ventilation (days), ICU length of stay (days) and Hospital length of stay (days), Mortality | NA |
| **Hacquin, 2021** | France  **1 center**  **67 patients** | Acute geriatric unit and Acute pulmonary care unit | March 2020 to January 2021 | ≥ 75 years old  +  COVID19 (confirmed)  +  AHRF  (respiratory rate superior to 30 breaths per minute, labored or paradoxical breathing, signs of hypercapnia, or difficulty talking) | In-hospital 30-day survival was the primary outcome.  Evaluation of comfort, anxiety, restlessness and dyspnea in patients treated by morphine or midazolam treated by HFNC. | NA |
| **Kabak, 2021** | Turkey  **1 center**  **54 patients** | Ward and ICU | July 2020 to August 2020 | COVID19 (confirmed)  +  Bilateral diffuse infiltration or ≥ 50% involvement of the total lung area in Computed Tomography, no need for invasive mechanical ventilation support at presentation,  SpO_2_ of < 88% despite maximum nasal oxygen support, mild and moderate ARDS, COVID-19 PCR (polymerase chain reaction) test positivity, D-dimer > 500 and ferritin > 500, or presence of cytokine storm | Intubation rate, ICU mortality ICU length of stay and treatment costs | NA |
| **Kamil, 2023** | Malaysia  **2 centers**  **110 patients** | Emergency departments | June to August 2021 | COVID19 (confirmed)  +  AHRF (confirmed COVID-19 pneumonia with respiratory rate > 30 breaths/min, severe respiratory distress, or peripheral oxygen saturation < 90% on room air) | Improvement of partial pressure arterial oxygen (PaO_2_) at two hours. Intubation rate, ventilator-free days, hospital length of stay, and 28-day mortality. | NA |
| **Liao, 2020** | China  **21 centers**  **63 patients** | ICU | January to March 2020 | COVID19 (confirmed)  +  AHRF (confirmed COVID-19 patients who met any of the five following criteria were included as severe cases:  1) dyspnea or respiratory frequency ≥30 breaths/minute;  2) pulse oxygen saturation (SPO_2_) ≤93% without oxygen therapy in resting state;  3) PaO_2_:FiO_2_ ratio < 300 mmHg;  4) lung infiltrates >50% within 24–48 hours;  5) respiratory failure, septic shock, and/or multiple organ dysfunction) | Clinical outcomes by D28, including rapid recovery (RR), prolonged recovery (PR) and no recovery (NR), were defined as follows.  1) RR: patient fully meeting the discharge criteria before D28, with normal body temperature ≥3 days, obvious improvement in respiratory symptoms and pulmonary imaging, and twice-negative nucleic acid tests (sampling interval being at least 24 hours) on respiratory samples;  2) PR: patient partially meeting the discharge criteria on D28 and still requiring hospitalization but without advanced respiratory support; 3) NR: death or the patient still in need of advanced respiratory support on D28. | NA |
| **Sayan, 2021** | Turkey  **1 center**  **43 patients** | ICU | March to May 2020 | COVID19 (confirmed)  +  Diagnosis of pneumonia (clinical findings and appearance of multifocal ground-glass opacities that had consolidated on computed tomography)  +  AHRF (Acute respiratory failure was defined as having a PaO_2_:FiO_2_ ratio < 300 mm Hg despite conventional oxygen therapy with a reservoir mask of 6 L/min) | Intubation requirement, intensive care length of stay, and short-term mortality | NA |
| **Wendel‑Garcia, 2022**  AHRF: acute hypoxemic respiratory failure; COT: conventional oxygen therapy; FiO_2_: fraction of inspired oxygen; HFNC: high-flow nasal cannula therapy; ICU: intensive care unit; IMV: invasive mechanical ventilation; NIV: noninvasive ventilation; PaO_2_: arterial partial pressure of oxygen; SpO_2_: oxygen saturation as measured by pulse oximetry | Spain  **26 centers**  **992 patients** | ICU | March to April 2020 | COVID19 (confirmed)  +  Clinical signs and symptoms compatible with COVID-19 pneumonia, bilateral infiltrates in the chest X-ray, need for supplemental oxygen to keep arterial oxygen saturation measured with a pulse oximeter (SpO_2_) above 90%, | Intubation rate, Mortality rate, Ventilator-free days, ICU length of stay | Decisions regarding intubation of the trachea were based on clinical grounds and judgment of the intensivist in charge. |
