# Supplementary material for: High-flow nasal cannula oxygen versus conventional oxygen therapy for acute respiratory failure due to COVID-19: a systematic review and meta-analysis
Source: Ann Intensive Care. 2023 Nov 23;13:114. doi: 10.1186/s13613-023-01208-8 (PMC10667189; doi:10.1186/s13613-023-01208-8)
Supplement: Supplementary file 1 — Additional file 1: Figure S1. Risk of bias graph (ROB 2) for intubation outcome from randomized controlled trials. Figure S2. Funnel plot for intubation rate and assessment of small-study effects by Rücker’s limit meta-analysis method using Arcsine difference and Peters arcsine test. Figure S3. Funnel plot for mortality rate and assessment of small-study effects by Rücker’s limit meta-analysis method using arcsine difference and Peters arcsine test. Figure S4. Forest plot of intubation rate comparison between HFNC and COT from prospective and retrospective studies (random-effects meta-analysis by the Mantel–Haenszel method). COT, conventional oxygen therapy; HFNC, high-flow nasal cannula; M-H, Mantel–Haenszel. Figure S5. Sensitivity analysis of the risk of intubation through the leave-one-out strategy for the randomized controlled trials (fixed-effects meta-analysis by the Mantel–Haenszel method). COT, conventional oxygen therapy; HFNC, high-flow nasal cannula. Figure S6. Sensitivity analysis of the risk of intubation through the leave-one-out strategy for all studies (random-effects meta-analysis by the Mantel–Haenszel method). COT, conventional oxygen therapy; HFNC, high-flow nasal cannula. Figure S7. Forest plot of intubation rate comparison between HFNC and COT from randomized controlled trials according to the location of admission (random-effects meta-analysis by the Mantel–Haenszel method). COT, conventional oxygen therapy; HFNC, high-flow nasal cannula; ICU, intensive care unit; M-H, Mantel–Haenszel. Figure S8. Forest plot of mortality comparison between HFNC and COT from prospective and retrospective studies (random-effects meta-analysis by the Mantel–Haenszel method). COT, conventional oxygen therapy; HFNC, high-flow nasal cannula; M-H, Mantel–Haenszel. Figure S9. Forest plot of mortality rate comparison between HFNC and COT from randomized controlled trials according to the location of admission (fixed-effects meta-analysis by the Mantel–Haenszel method). [file 13613_2023_1208_MOESM1_ESM.zip › Supplementary/Supplementary table S4.docx]

**Supplementary table S4. Patient characteristics of the non-randomized controlled trials.**

1. **Prospective observational studies**

| **Study** | **Group**  **(N)** | **Age, y** | **Sex**  **(Male, n, %)** | **BMI**  **(kg/m2)** | **Severity score (admission)** | **Baseline**  **oxygenation level** | **Respiratory rate, /min** | **FiO_2_** | **Flow rate (L/min)** | **Proning (n, %)** | **Corticosteroids (n, %)** |
| --- | --- | --- | --- | --- | --- | --- | --- | --- | --- | --- | --- |
| **COVID-ICU group, 2021** | HFNC  (567) | 63 ± 13 | 554 (72) | 28±4  ≥ 30 kg/m2  179 (35%) | SOFA  3 ± 1 | PaO_2_:FiO_2_  108 ± 42 | NA | 0.76 ± 0.23 | 50 ± 15 | NA | 85 (15) |
|  | COT  (766) | 62 ± 13 | 427 (75) | 29 ± 5  ≥ 30 kg/m2  260 (37%) | SOFA  3 ± 1 | PaO_2_:FiO_2_  155 ± 74 | NA | NA | 8 ± 6 | NA | 50 (7) |
| **Roger, 2021** | HFNC  (179) | (All patients)  65 ± 12) | NA | NA | NA | (All patients)  PaO_2_:FiO_2_  128 ± 69 | (All patients)  32 ± 31 | (All patients)  0.76 ± 0.18 | NA | NA | (All patients)  212 (22) |
|  | COT  (228) | (All patients)  65 ± 12 | NA | NA | NA | (All patients)  PaO_2_:FiO_2_  128 ± 69 | (All patients)  32 ± 31 | (All patients)  0.76 ± 0.18 | NA | NA | (All patients)  212 (22) |
| **Wendel-Garcia, 2021** | HFNC  (87) | 66 ± 15 | 65 (75) | 29 ± 5 | SOFA  6 ± 1 | PaO_2_:FiO_2_  123 ± 55 | 28 ± 8 | 0.65 ± 0.29 | NA | 31 (68) | 13 (29) |
|  | COT  (85) | 62 ± 14 | 63 (75) | 29 ± 5 | SOFA  6 ± 1 | PaO_2_:FiO_2_  133 ± 44 | 28 ± 6 | 0.60 ± 0.00 | NA | 35 (64) | 11 (20) |
| **ACCCOS, 2021** | HFNC  (589) | NA | NA | NA | NA | NA | NA | NA | NA | NA | NA |
|  | COT  (1352) | NA | NA | NA | NA | NA | NA | NA | NA | NA | NA |
| **Hansen, 2021** | HFNC  (30) | 69 ± 13 | 21 (70) | 32 ± 8 | SOFA  7 ± 2 | PaO_2_:FiO_2_  152 ± 65 | NA | NA | NA | NA | NA |
|  | COT  (61) | 68 ± 12 | 37 (60) | 31 ± 10 | SOFA  8 ± 3 | PaO_2_:FiO_2_  152 ± 65 | NA | NA | NA | NA | NA |

Categorical data are expressed as number of patients (percentage of total group size). Continuous data are expressed as mean ± standard deviation. BMI: body mass index; COT: conventional oxygen therapy; FiO_2_: fraction of inspired oxygen; HFNC: high-flow nasal cannula therapy; NA: not available data; PaO_2_: arterial partial pressure of oxygen; SpO_2_: oxygen saturation as measured by pulse oximetry

1. **Retrospective observational studies**

| **Study** | **Group**  **(N)** | **Age, y** | **Sex**  **(Male, n, %)** | **BMI**  **(kg/m2)** | **Severity score (admission)** | **Baseline**  **oxygenation level** | **Respiratory rate, /min** | **FiO_2_** | **Flow rate (L/min)** | **Use of proning (n, %)** | **Use of**  **steroids (n, %)** |
| --- | --- | --- | --- | --- | --- | --- | --- | --- | --- | --- | --- |
| **Bonnet, 2021** | HFNC  (76) | 59 ± 12 | 62 (82) | 29 ± 6 | SAPS II  39 ± 16 | PaO_2_  73 ± 15 | 32 ± 6 | NA | NA | NA | 41 (54) |
|  | COT  (62) | 58 ± 12 | 50 (81) | 29 ± 5 | SAPS II  37 ± 15 | PaO_2_  75 ± 17 | 31 ± 7 | NA | 10 ± 8 | NA | 25 (40) |
| **Demoule 2020** | HFNC  (146) | 60 ± 10 | 115 (79) | 28 ± 4 | SOFA  4 ± 2 | PaO_2_:FiO_2_  154 ± 102 | 28 ± 5 | 0.96 ± 0.07 | NA | NA | NA |
|  | COT  (233) | 60 ± 11 | 176 (75) | 29 ± 5 | SOFA  6 ± 3 | PaO_2_:FiO_2_  156 ± 89 | 31 ± 6 | 0.59 ± 0.11 | 13 ± 3 | NA | NA |
| **Gallardo, 2022** | HFNC  (42) | 63 ± 11 | 34 (81) | NA | SOFA  2 ± 1 | SpO_2_ at room air  92 ± 5 | 22 ± 3 | NA | NA | NA | 41 (98) |
|  | COT  (42) | 63 ± 11 | 34 (81) | NA | SOFA  3 ± 1 | SpO_2_ at room air  92 ± 5 | 24 ± 6 | NA | NA | NA | 40 (95) |
| **Hacquin, 2021** | HFNC  (41) | 88 ± 4 | 27 (66) | NA | NA | SpO_2_:FiO_2_  104 ± 25 | 37 ± 28 | NA | NA | NA | 37 (90) |
|  | COT  (26) | 87 ± 4 | 14 (54) | NA | NA | SpO_2_:FiO_2_  128 ± 27 | 30 ± 8 | NA | NA | NA | 15 (60) |
| **Kabak, 2021** | HFNC  (26) | 62 ± 16 | 19 (73) | NA | SOFA  9 ± 4 | PaO_2_:FiO_2_  189 ± 24 | NA | NA | NA | NA | 26 (100) |
|  | COT  (28) | 65 ± 13 | 17 (61) | NA | SOFA  10 ± 4 | PaO_2_:FiO_2_  190 ± 29 | NA | NA | NA | NA | 28 (100) |
| **Kamil, 2023** | HFNC  (58) | 51 ± 15 | 38 (66) | NA | SOFA  2 ± 1 | PaO_2_:FiO_2_  72 ± 20 | 32 ± 6 | 0.58 ± 0.04 | 58 ± 4 | 12 (21) | 58 (100) |
|  | COT  (52) | 48 ± 18 | 33 (64) | NA | SOFA  5 ± 5 | PaO_2_:FiO_2_  67 ± 9 | 38 ± 2 | 1.00 ± 0.00 | 20 ± 0 | 46 (89) | 52 (100) |
| **Liao, 2020** | HFNC  (8) | (All patients)  54 ± 21 | (All patients)  51 (63) | (All patients)  25 ± 4 | SOFA  (All patients)  4 ± 2 | PaO_2_:FiO_2_  (All patients) 221 ± 90 | (All patients) 23 ± 4 | (All patients)  0.35 ± 0.09 | NA | (All patients)  16 (20) | (All patients)  44 (54) |
|  | COT  (55) | (All patients)  54 ± 21 | (All patients)  51 (63) | (All patients)  25 ± 4 | SOFA  (All patients)  4 ± 2 | PaO_2_:FiO_2_  (All patients) 221 ± 90 | (All patients) 23 ± 4 | (All patients)  0.35 ± 0.09 | NA | (All patients)  16 (20) | (All patients)  44 (54) |
| **Sayan, 2021** | HFNC  (24) | 63 ± 12 | 17 (71) | 27 ± 3 | NA | PaO_2_:FiO_2_  171 ± 19 | 33 ± 5 | NA | NA | 0 (0) | NA |
|  | COT  (19) | 70 ± 12 | 13 (68) | 27 ± 3 | NA | PaO_2_:FiO_2_  184 ± 40 | 33 ± 4 | NA | NA | 0 (0) | NA |
| **Wendel‑**  **Garcia, 2022** | HFNC  (439) | 62 ± 12 | 297 (68) | 29 ± 4 | NA | PaO_2_:FiO_2_  183 ± 76  (after intubation) | 23 ± 4  (after intubation) | 0.60 ± 0.15  (after intubation) | NA | NA | NA |
|  | COT  (553) | 62 ± 11 | 374 (68) | 28 ± 4 | NA | PaO_2_:FiO_2_  193 ± 95  (after intubation) | 23 ± 4  (after intubation) | 0.67 ± 0.24  (after intubation) | NA | NA | NA |

Categorical data are expressed as number of patients (percentage of total group size). Continuous data are expressed as mean ± standard deviation. BMI: body mass index; COT: conventional oxygen therapy; FiO_2_: fraction of inspired oxygen; HFNC: high-flow nasal cannula therapy; NA: not available data; PaO_2_: arterial partial pressure of oxygen; SpO_2_: oxygen saturation as measured by pulse oximetry
