# Supplementary material for: High-flow nasal cannula oxygen versus conventional oxygen therapy for acute respiratory failure due to COVID-19: a systematic review and meta-analysis
Source: Ann Intensive Care. 2023 Nov 23;13:114. doi: 10.1186/s13613-023-01208-8 (PMC10667189; doi:10.1186/s13613-023-01208-8)
Supplement: Supplementary file 1 — Additional file 1: Figure S1. Risk of bias graph (ROB 2) for intubation outcome from randomized controlled trials. Figure S2. Funnel plot for intubation rate and assessment of small-study effects by Rücker’s limit meta-analysis method using Arcsine difference and Peters arcsine test. Figure S3. Funnel plot for mortality rate and assessment of small-study effects by Rücker’s limit meta-analysis method using arcsine difference and Peters arcsine test. Figure S4. Forest plot of intubation rate comparison between HFNC and COT from prospective and retrospective studies (random-effects meta-analysis by the Mantel–Haenszel method). COT, conventional oxygen therapy; HFNC, high-flow nasal cannula; M-H, Mantel–Haenszel. Figure S5. Sensitivity analysis of the risk of intubation through the leave-one-out strategy for the randomized controlled trials (fixed-effects meta-analysis by the Mantel–Haenszel method). COT, conventional oxygen therapy; HFNC, high-flow nasal cannula. Figure S6. Sensitivity analysis of the risk of intubation through the leave-one-out strategy for all studies (random-effects meta-analysis by the Mantel–Haenszel method). COT, conventional oxygen therapy; HFNC, high-flow nasal cannula. Figure S7. Forest plot of intubation rate comparison between HFNC and COT from randomized controlled trials according to the location of admission (random-effects meta-analysis by the Mantel–Haenszel method). COT, conventional oxygen therapy; HFNC, high-flow nasal cannula; ICU, intensive care unit; M-H, Mantel–Haenszel. Figure S8. Forest plot of mortality comparison between HFNC and COT from prospective and retrospective studies (random-effects meta-analysis by the Mantel–Haenszel method). COT, conventional oxygen therapy; HFNC, high-flow nasal cannula; M-H, Mantel–Haenszel. Figure S9. Forest plot of mortality rate comparison between HFNC and COT from randomized controlled trials according to the location of admission (fixed-effects meta-analysis by the Mantel–Haenszel method). [file 13613_2023_1208_MOESM1_ESM.zip › Supplementary/Supplementary table S5.docx]

**Supplementary table S5. Pre-defined intubation criteria of the randomized controlled trials**

| **Study, year** | **Pre-defined criteria for intubation** |
| --- | --- |
| **Bouadma, 2022** | Any of the three pre-specified criteria:  (1) signs of persisting or worsening respiratory failure, defined by at least two of the following criteria: a respiratory rate above 35 cycles/min, lack of improvement of signs of respiratory-muscle fatigue, development of copious tracheal secretions, acidosis with a pH below 7.35, SpO_2_ below 90% despite FiO_2_ ≥80% for more than five min without technical dysfunction, or intolerance to NIV;  (2) hemodynamic instability defined by systolic blood pressure below 90 mm Hg, mean blood pressure below 65 mm Hg or vasopressor requirement; or (3) deterioration of neurologic status with a Glasgow coma scale below 12. |
| **Crimi, 2022** | Predefined criteria for considering the escalation of respiratory support to CPAP, NIV or IMV were the presence of SpO_2_ ≤92% despite COT or HFNO or PaO2:FiO_2_ ratio ≤180 mm Hg with FiO_2_ ≥50%, and at least one of the following: respiratory rate ≥28 breaths/min, severe dyspnea, signs of increased work of breathing (*e.g.*, use of accessory muscles).  The type of respiratory support chosen for escalation was selected by treating physicians based on their clinical judgement. Escalation of respiratory support could be performed in the hospital ward where the patient was admitted or after being transferred to the intensive care unit (ICU). |
| **Frat, 2022** | Pre-determined criteria for endotracheal intubation and IMV: 1. signs of persisting or worsening respiratory failure, defined by at least two of the following criteria: respiratory rate above 40 cycles/min, appearance or worsening of signs of respiratory-muscle fatigue, acidosis (metabolic or respiratory) with pH below 7.35, life-threatening hypoxemia defined as need of O2 flow equal to or above 15L/min or of FiO_2_ ≥ 80% to maintain a SpO_2_ ≥ 92%, or PaO2:FiO_2_ ratio <100 mm Hg.  2. threatening hypoxemia: recurrent episodes of SpO_2_ <80% or persisting SpO_2_ <88% with maximal oxygen support.  3. Or one of the following: hemodynamic instability with signs of hypoperfusion, cardiac arrest; deterioration of neurologic status with Glasgow coma scale below 12 points or agitation. |
| **Nazir, 2022** | 1. Persistent respiratory distress: RR > 40 breaths/ min, signs of labored breathing, use of accessory muscles of respiration.  2. Copious airway secretions.  3. ABG: metabolic/respiratory acidosis, pH <7.25, PaO_2_ <55 mm Hg, PaCO_2_ > 55 mm Hg.  4. SpO_2_ <90% on current oxygen delivery device.  5. Signs of hemodynamic instability - MAP < 60 mm Hg, requirement of ionotropic support (norepinephrine >0.10 µgr.kg.min-1) with normal CVP, CRT >10 seconds, lactate ≥4.0 mmol/L  Neurological impairment (GCS ≤8) |
| **Ospina-Tascon, 2021** | • Signs of persistent respiratory distress: Respiratory rate > 40 / min, No improvement of laborious breathing, use of accessory muscles, Development of copious bronchial secretions / impossibility to manage bronchial secretions, Acidosis (metabolic / respiratory): pH < 7.25 - PaO_2_ < 55 mm Hg - PaCO_2_ > 55 mm Hg, SpO_2_ < 92% for more than five minutes (discarding signal problems or other technical issues)  • Signs of hemodynamic derangement: Persistent systolic arterial pressure < 90 or mean arterial pressure < 60 mm Hg, with vasopressor support requirement (norepinephrine > 0.10 μgr.kg.min-1) in presence of an adequate intravascular volume, Clinical signs of severe tissue hypoperfusion: capillary refill time > 10 seconds; Mottling score ≥ 4 - Arterial lactate ≥ 4.0 mmol/L in presence of any clinical sign of tissue hypoperfusion (capillary refill time > 3 seconds; Mottling score ≥ 2)  • Signs of neurological derangement - Neurological impairment (Glasgow coma scale ≤ 12) |
| **Perkins, 2022** | Tracheal intubation was carried out at the clinician’s judgement. |
| **Teng, 2020** | Patients requiring intubation were eliminated from the analysis |

ABG: arterial blood gas, COT: conventional oxygen therapy; CRT: central refilling time; FiO_2_: fraction of inspired oxygen; GCS: Glasgow coma scale; HFNO: high-flow nasal oxygen; IMV: invasive mechanical ventilation; MAP: mean arterial pressure; NIV: noninvasive ventilation; PaCO_2_: arterial partial pressure of carbon dioxide; PaO_2_: arterial partial pressure of oxygen; RR: respiratory rate; SpO_2_: oxygen saturation as measured by pulse oximetry.
