# Supplementary material for: High-flow nasal cannula oxygen versus conventional oxygen therapy for acute respiratory failure due to COVID-19: a systematic review and meta-analysis
Source: Ann Intensive Care. 2023 Nov 23;13:114. doi: 10.1186/s13613-023-01208-8 (PMC10667189; doi:10.1186/s13613-023-01208-8)
Supplement: Supplementary file 1 — Additional file 1: Figure S1. Risk of bias graph (ROB 2) for intubation outcome from randomized controlled trials. Figure S2. Funnel plot for intubation rate and assessment of small-study effects by Rücker’s limit meta-analysis method using Arcsine difference and Peters arcsine test. Figure S3. Funnel plot for mortality rate and assessment of small-study effects by Rücker’s limit meta-analysis method using arcsine difference and Peters arcsine test. Figure S4. Forest plot of intubation rate comparison between HFNC and COT from prospective and retrospective studies (random-effects meta-analysis by the Mantel–Haenszel method). COT, conventional oxygen therapy; HFNC, high-flow nasal cannula; M-H, Mantel–Haenszel. Figure S5. Sensitivity analysis of the risk of intubation through the leave-one-out strategy for the randomized controlled trials (fixed-effects meta-analysis by the Mantel–Haenszel method). COT, conventional oxygen therapy; HFNC, high-flow nasal cannula. Figure S6. Sensitivity analysis of the risk of intubation through the leave-one-out strategy for all studies (random-effects meta-analysis by the Mantel–Haenszel method). COT, conventional oxygen therapy; HFNC, high-flow nasal cannula. Figure S7. Forest plot of intubation rate comparison between HFNC and COT from randomized controlled trials according to the location of admission (random-effects meta-analysis by the Mantel–Haenszel method). COT, conventional oxygen therapy; HFNC, high-flow nasal cannula; ICU, intensive care unit; M-H, Mantel–Haenszel. Figure S8. Forest plot of mortality comparison between HFNC and COT from prospective and retrospective studies (random-effects meta-analysis by the Mantel–Haenszel method). COT, conventional oxygen therapy; HFNC, high-flow nasal cannula; M-H, Mantel–Haenszel. Figure S9. Forest plot of mortality rate comparison between HFNC and COT from randomized controlled trials according to the location of admission (fixed-effects meta-analysis by the Mantel–Haenszel method). [file 13613_2023_1208_MOESM1_ESM.zip › Supplementary/Supplementary table S6_corrected.docx]

**Supplementary table S6. GRADE evidence profile for the studies in the meta-analysis.**

Population: Adult patients with acute hypoxemic respiratory failure du to COVID-19

Intervention: High-flow nasal cannula therapy (HFNC)

Comparator: Conventional oxygen therapy (COT)

| **Outcome**  Timeframe | **Study results and measurements** | **Absolute effect estimates** | | **Certainty of the Evidence**  (Quality of evidence) | **Plain language summary** |
| --- | --- | --- | --- | --- | --- |
|  |  | COT | HFNC |  |  |
| **Intubation** | Relative risk: 0.89  (CI 95% 0.80 to 0.98)  Based on data from 1227 participants in 6 studies  Follow up 28 days | **370**  per 1000 | **329**  per 1000 | **High**^1^ | HFNC decreases intubation requirement |
|  |  | Difference: **41 fewer per 1000**  (CI 95% 74 fewer to 7 fewer) | |  |  |
| **Mortality** | Relative risk: 0.93  (CI 95% 0.77 to 1.11)  Based on data from 1228 participants in 6 studies  Follow up 28 days | **180**  per 1000 | **167**  per 1000 | **High** | HFNC has little or no difference on mortality at day 28 |
|  |  | Difference: **13 fewer per 1000**  (CI 95% 41 fewer to 20 more) | |  |  |
| **Length of stay in hospital** | Measured by:  Scale: - Lower better  Based on data from 1157 participants in 5 studies  Follow up 28 days | **17.8**  days (Mean) | **16.5**  days (Mean) | **Moderate**  Due to serious inconsistency^2^ | HFNC decreases length of stay in hospital |
|  |  | Difference: **MD 1.21 fewer**  (CI 95% 2.34 fewer to 0.07 fewer) | |  |  |
| **Length of stay in intensive care unit** | Measured by:  Scale: - Lower better  Based on data from 1159 participants in 5 studies  Follow up 28 days | **12.3**  days (Mean) | **10.6**  days (Mean) | **High**^3^ | HFNC probably has little or no difference on length of stay in intensive care unit |
|  |  | Difference: **MD 1.34 fewer**  (CI 95% 2.86 fewer to 0.19 more) | |  |  |

1. **Inconsistency: no serious.** The direction of the effect is not consistent between the included studies;
2. **Inconsistency: serious.** The direction of the effect is not consistent between the included studies, The magnitude of statistical heterogeneity was high, with I^2: 51 %.; **Imprecision: no serious.** Wide confidence intervals;
3. **Inconsistency: no serious.** The direction of the effect is not consistent between the included studies; **Imprecision: no serious.** Wide confidence intervals;
