# Supplementary figures and images for: High-flow nasal cannula oxygen versus conventional oxygen therapy for acute respiratory failure due to COVID-19: a systematic review and meta-analysis
Source: Ann Intensive Care. 2023 Nov 23;13:114. doi: 10.1186/s13613-023-01208-8 (PMC10667189; doi:10.1186/s13613-023-01208-8)

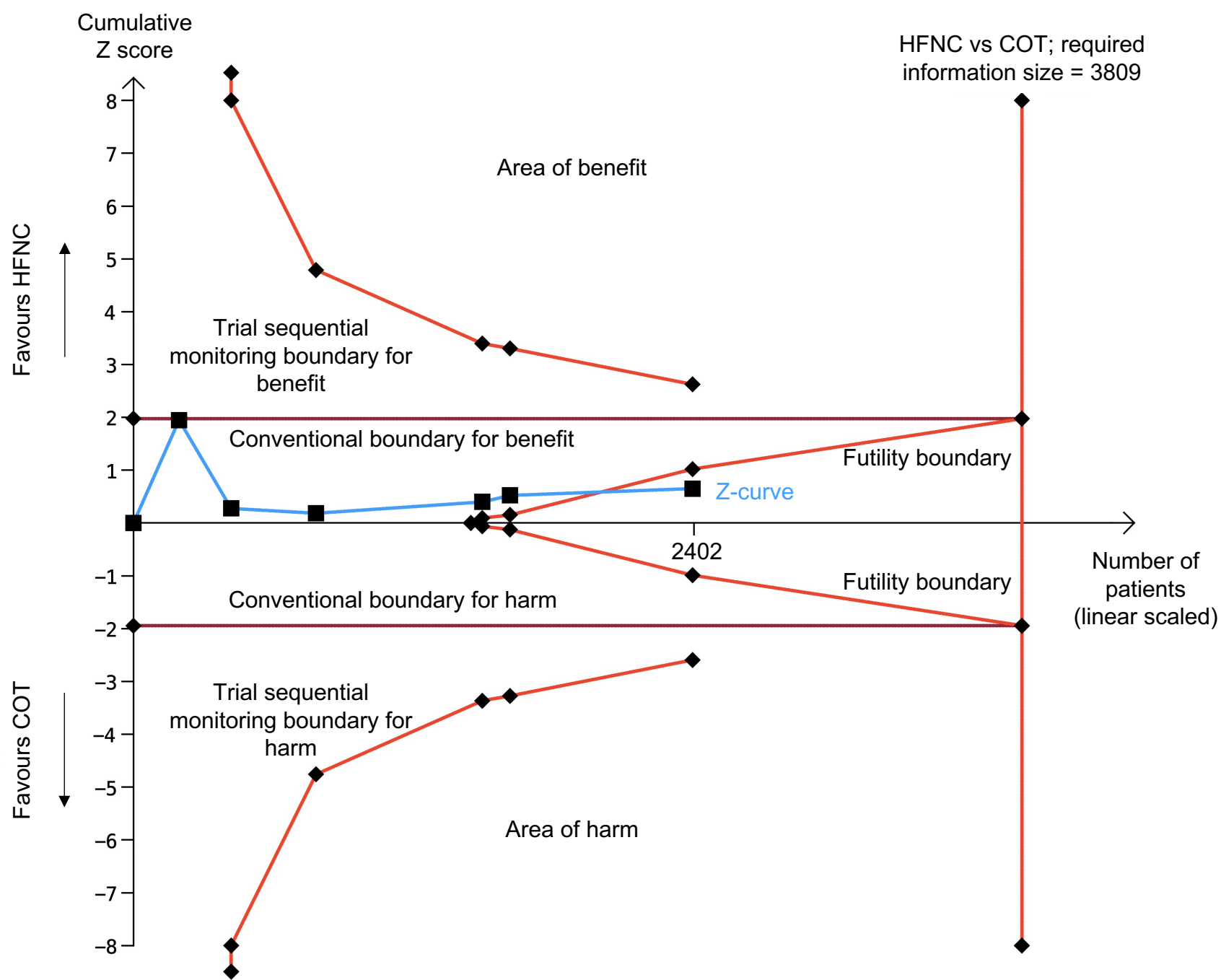

Supplement: Supplementary file 1 — Additional file 1: Figure S1. Risk of bias graph (ROB 2) for intubation outcome from randomized controlled trials. Figure S2. Funnel plot for intubation rate and assessment of small-study effects by Rücker’s limit meta-analysis method using Arcsine difference and Peters arcsine test. Figure S3. Funnel plot for mortality rate and assessment of small-study effects by Rücker’s limit meta-analysis method using arcsine difference and Peters arcsine test. Figure S4. Forest plot of intubation rate comparison between HFNC and COT from prospective and retrospective studies (random-effects meta-analysis by the Mantel–Haenszel method). COT, conventional oxygen therapy; HFNC, high-flow nasal cannula; M-H, Mantel–Haenszel. Figure S5. Sensitivity analysis of the risk of intubation through the leave-one-out strategy for the randomized controlled trials (fixed-effects meta-analysis by the Mantel–Haenszel method). COT, conventional oxygen therapy; HFNC, high-flow nasal cannula. Figure S6. Sensitivity analysis of the risk of intubation through the leave-one-out strategy for all studies (random-effects meta-analysis by the Mantel–Haenszel method). COT, conventional oxygen therapy; HFNC, high-flow nasal cannula. Figure S7. Forest plot of intubation rate comparison between HFNC and COT from randomized controlled trials according to the location of admission (random-effects meta-analysis by the Mantel–Haenszel method). COT, conventional oxygen therapy; HFNC, high-flow nasal cannula; ICU, intensive care unit; M-H, Mantel–Haenszel. Figure S8. Forest plot of mortality comparison between HFNC and COT from prospective and retrospective studies (random-effects meta-analysis by the Mantel–Haenszel method). COT, conventional oxygen therapy; HFNC, high-flow nasal cannula; M-H, Mantel–Haenszel. Figure S9. Forest plot of mortality rate comparison between HFNC and COT from randomized controlled trials according to the location of admission (fixed-effects meta-analysis by the Mantel–Haenszel method). [file 13613_2023_1208_MOESM1_ESM.zip › Supplementary/Supplementary figure S10. TSA of mortality outcome.pdf]

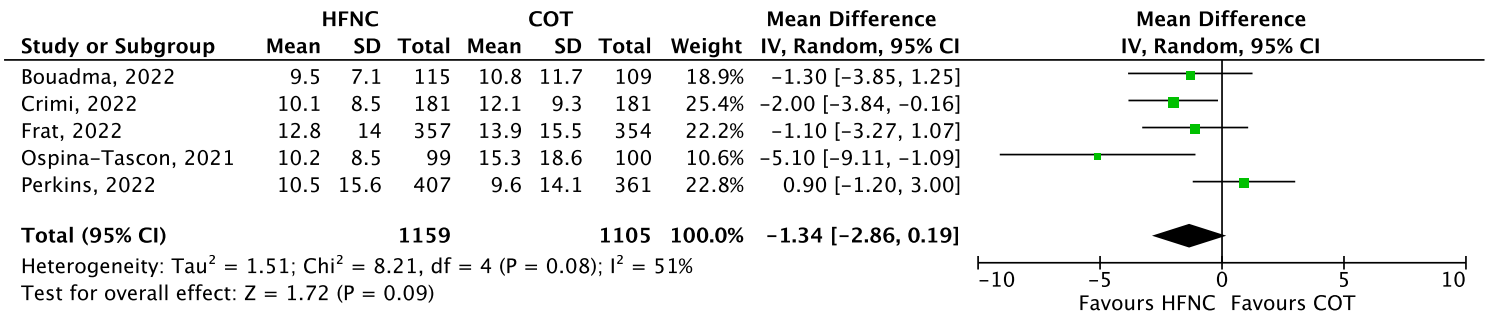

Supplement: Supplementary file 1 — Additional file 1: Figure S1. Risk of bias graph (ROB 2) for intubation outcome from randomized controlled trials. Figure S2. Funnel plot for intubation rate and assessment of small-study effects by Rücker’s limit meta-analysis method using Arcsine difference and Peters arcsine test. Figure S3. Funnel plot for mortality rate and assessment of small-study effects by Rücker’s limit meta-analysis method using arcsine difference and Peters arcsine test. Figure S4. Forest plot of intubation rate comparison between HFNC and COT from prospective and retrospective studies (random-effects meta-analysis by the Mantel–Haenszel method). COT, conventional oxygen therapy; HFNC, high-flow nasal cannula; M-H, Mantel–Haenszel. Figure S5. Sensitivity analysis of the risk of intubation through the leave-one-out strategy for the randomized controlled trials (fixed-effects meta-analysis by the Mantel–Haenszel method). COT, conventional oxygen therapy; HFNC, high-flow nasal cannula. Figure S6. Sensitivity analysis of the risk of intubation through the leave-one-out strategy for all studies (random-effects meta-analysis by the Mantel–Haenszel method). COT, conventional oxygen therapy; HFNC, high-flow nasal cannula. Figure S7. Forest plot of intubation rate comparison between HFNC and COT from randomized controlled trials according to the location of admission (random-effects meta-analysis by the Mantel–Haenszel method). COT, conventional oxygen therapy; HFNC, high-flow nasal cannula; ICU, intensive care unit; M-H, Mantel–Haenszel. Figure S8. Forest plot of mortality comparison between HFNC and COT from prospective and retrospective studies (random-effects meta-analysis by the Mantel–Haenszel method). COT, conventional oxygen therapy; HFNC, high-flow nasal cannula; M-H, Mantel–Haenszel. Figure S9. Forest plot of mortality rate comparison between HFNC and COT from randomized controlled trials according to the location of admission (fixed-effects meta-analysis by the Mantel–Haenszel method). [file 13613_2023_1208_MOESM1_ESM.zip › Supplementary/Supplementary figure S11_ICU_LOS_wo_Teng.pdf]

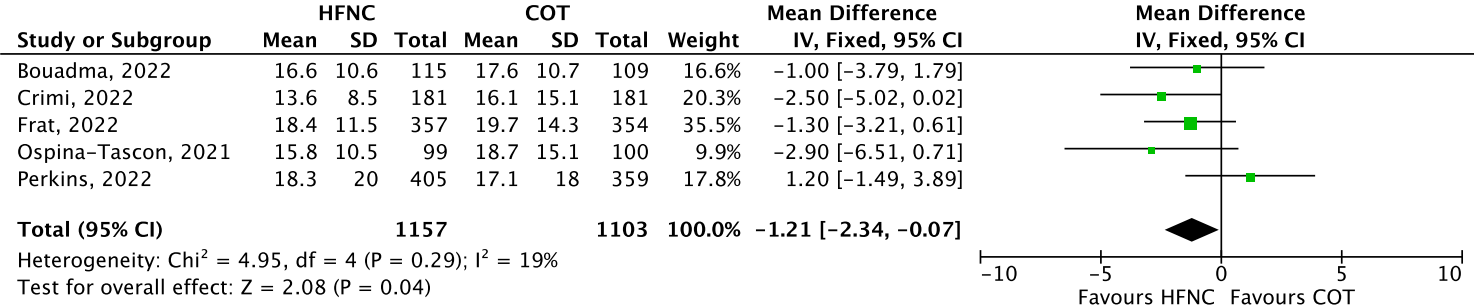

Supplement: Supplementary file 1 — Additional file 1: Figure S1. Risk of bias graph (ROB 2) for intubation outcome from randomized controlled trials. Figure S2. Funnel plot for intubation rate and assessment of small-study effects by Rücker’s limit meta-analysis method using Arcsine difference and Peters arcsine test. Figure S3. Funnel plot for mortality rate and assessment of small-study effects by Rücker’s limit meta-analysis method using arcsine difference and Peters arcsine test. Figure S4. Forest plot of intubation rate comparison between HFNC and COT from prospective and retrospective studies (random-effects meta-analysis by the Mantel–Haenszel method). COT, conventional oxygen therapy; HFNC, high-flow nasal cannula; M-H, Mantel–Haenszel. Figure S5. Sensitivity analysis of the risk of intubation through the leave-one-out strategy for the randomized controlled trials (fixed-effects meta-analysis by the Mantel–Haenszel method). COT, conventional oxygen therapy; HFNC, high-flow nasal cannula. Figure S6. Sensitivity analysis of the risk of intubation through the leave-one-out strategy for all studies (random-effects meta-analysis by the Mantel–Haenszel method). COT, conventional oxygen therapy; HFNC, high-flow nasal cannula. Figure S7. Forest plot of intubation rate comparison between HFNC and COT from randomized controlled trials according to the location of admission (random-effects meta-analysis by the Mantel–Haenszel method). COT, conventional oxygen therapy; HFNC, high-flow nasal cannula; ICU, intensive care unit; M-H, Mantel–Haenszel. Figure S8. Forest plot of mortality comparison between HFNC and COT from prospective and retrospective studies (random-effects meta-analysis by the Mantel–Haenszel method). COT, conventional oxygen therapy; HFNC, high-flow nasal cannula; M-H, Mantel–Haenszel. Figure S9. Forest plot of mortality rate comparison between HFNC and COT from randomized controlled trials according to the location of admission (fixed-effects meta-analysis by the Mantel–Haenszel method). [file 13613_2023_1208_MOESM1_ESM.zip › Supplementary/Supplementary figure S12_Hospital_LOS_wo_Teng.pdf]

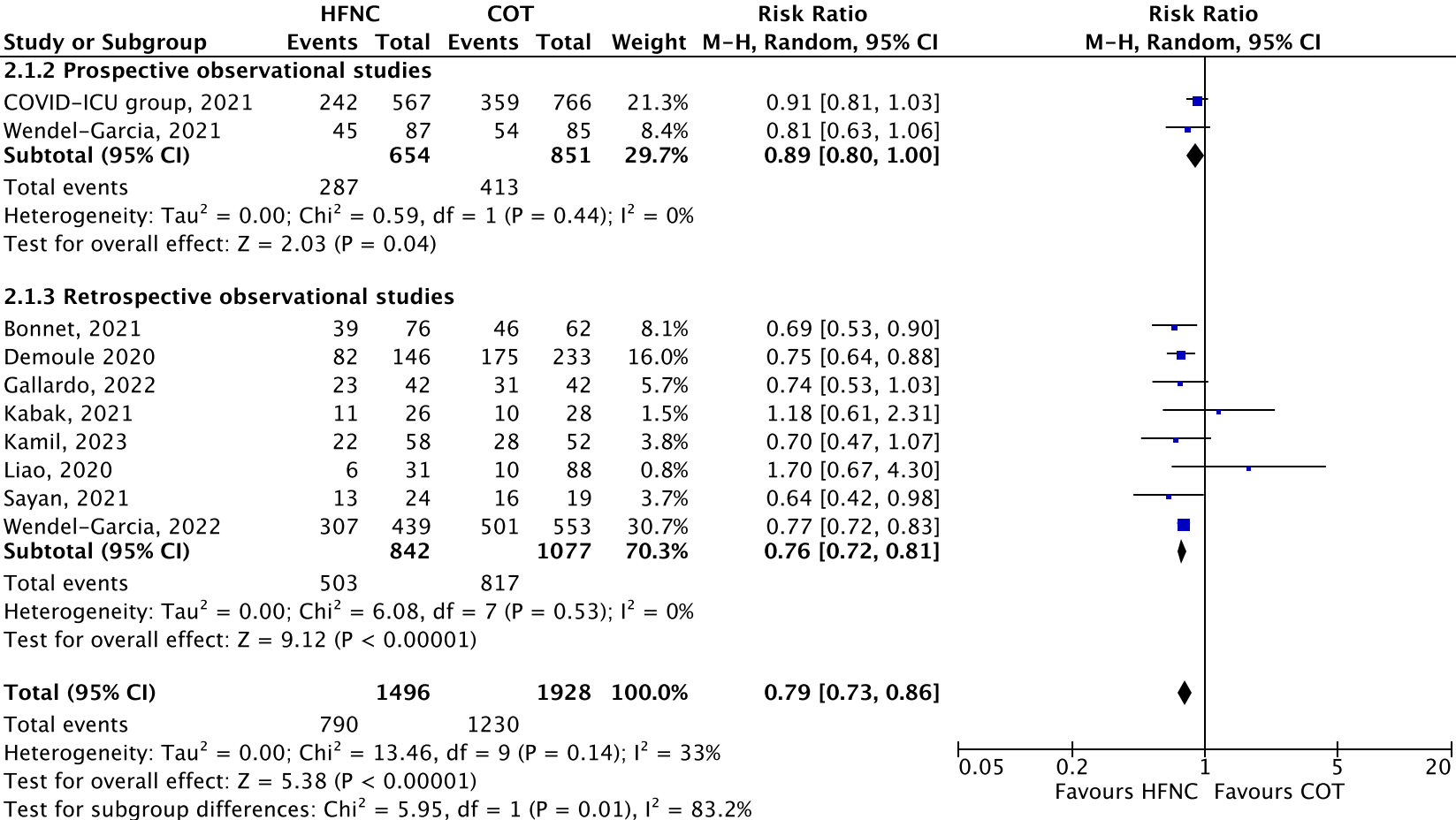

Supplement: Supplementary file 1 — Additional file 1: Figure S1. Risk of bias graph (ROB 2) for intubation outcome from randomized controlled trials. Figure S2. Funnel plot for intubation rate and assessment of small-study effects by Rücker’s limit meta-analysis method using Arcsine difference and Peters arcsine test. Figure S3. Funnel plot for mortality rate and assessment of small-study effects by Rücker’s limit meta-analysis method using arcsine difference and Peters arcsine test. Figure S4. Forest plot of intubation rate comparison between HFNC and COT from prospective and retrospective studies (random-effects meta-analysis by the Mantel–Haenszel method). COT, conventional oxygen therapy; HFNC, high-flow nasal cannula; M-H, Mantel–Haenszel. Figure S5. Sensitivity analysis of the risk of intubation through the leave-one-out strategy for the randomized controlled trials (fixed-effects meta-analysis by the Mantel–Haenszel method). COT, conventional oxygen therapy; HFNC, high-flow nasal cannula. Figure S6. Sensitivity analysis of the risk of intubation through the leave-one-out strategy for all studies (random-effects meta-analysis by the Mantel–Haenszel method). COT, conventional oxygen therapy; HFNC, high-flow nasal cannula. Figure S7. Forest plot of intubation rate comparison between HFNC and COT from randomized controlled trials according to the location of admission (random-effects meta-analysis by the Mantel–Haenszel method). COT, conventional oxygen therapy; HFNC, high-flow nasal cannula; ICU, intensive care unit; M-H, Mantel–Haenszel. Figure S8. Forest plot of mortality comparison between HFNC and COT from prospective and retrospective studies (random-effects meta-analysis by the Mantel–Haenszel method). COT, conventional oxygen therapy; HFNC, high-flow nasal cannula; M-H, Mantel–Haenszel. Figure S9. Forest plot of mortality rate comparison between HFNC and COT from randomized controlled trials according to the location of admission (fixed-effects meta-analysis by the Mantel–Haenszel method). [file 13613_2023_1208_MOESM1_ESM.zip › Supplementary/Supplementary Figure S4_IOT_Subgroup_Studies.pdf]

Sorted by Effect Size

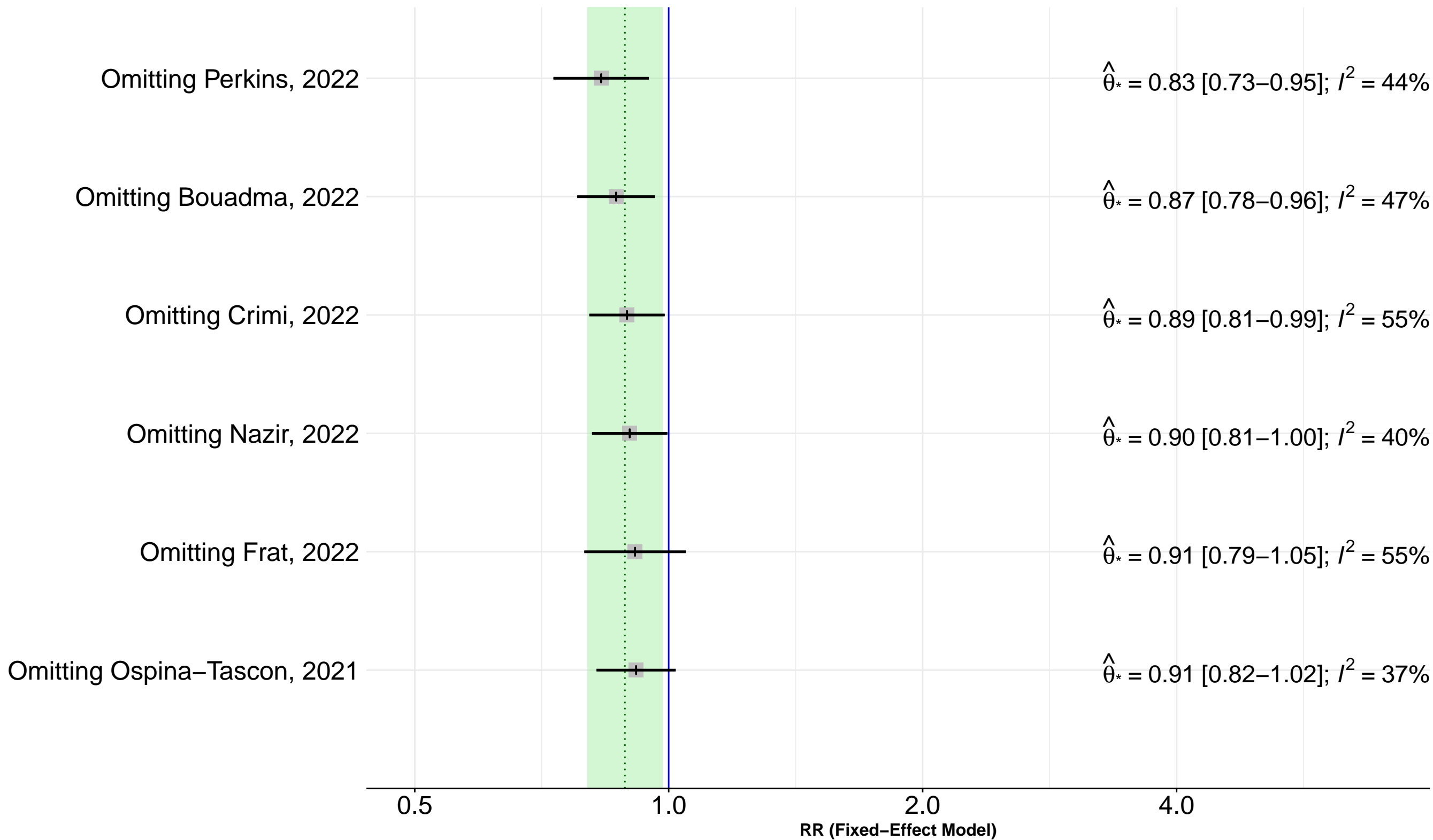

Supplement: Supplementary file 1 — Additional file 1: Figure S1. Risk of bias graph (ROB 2) for intubation outcome from randomized controlled trials. Figure S2. Funnel plot for intubation rate and assessment of small-study effects by Rücker’s limit meta-analysis method using Arcsine difference and Peters arcsine test. Figure S3. Funnel plot for mortality rate and assessment of small-study effects by Rücker’s limit meta-analysis method using arcsine difference and Peters arcsine test. Figure S4. Forest plot of intubation rate comparison between HFNC and COT from prospective and retrospective studies (random-effects meta-analysis by the Mantel–Haenszel method). COT, conventional oxygen therapy; HFNC, high-flow nasal cannula; M-H, Mantel–Haenszel. Figure S5. Sensitivity analysis of the risk of intubation through the leave-one-out strategy for the randomized controlled trials (fixed-effects meta-analysis by the Mantel–Haenszel method). COT, conventional oxygen therapy; HFNC, high-flow nasal cannula. Figure S6. Sensitivity analysis of the risk of intubation through the leave-one-out strategy for all studies (random-effects meta-analysis by the Mantel–Haenszel method). COT, conventional oxygen therapy; HFNC, high-flow nasal cannula. Figure S7. Forest plot of intubation rate comparison between HFNC and COT from randomized controlled trials according to the location of admission (random-effects meta-analysis by the Mantel–Haenszel method). COT, conventional oxygen therapy; HFNC, high-flow nasal cannula; ICU, intensive care unit; M-H, Mantel–Haenszel. Figure S8. Forest plot of mortality comparison between HFNC and COT from prospective and retrospective studies (random-effects meta-analysis by the Mantel–Haenszel method). COT, conventional oxygen therapy; HFNC, high-flow nasal cannula; M-H, Mantel–Haenszel. Figure S9. Forest plot of mortality rate comparison between HFNC and COT from randomized controlled trials according to the location of admission (fixed-effects meta-analysis by the Mantel–Haenszel method). [file 13613_2023_1208_MOESM1_ESM.zip › Supplementary/Supplementary figure S5-LeaveOneOut_RCTs_Intubation.pdf]

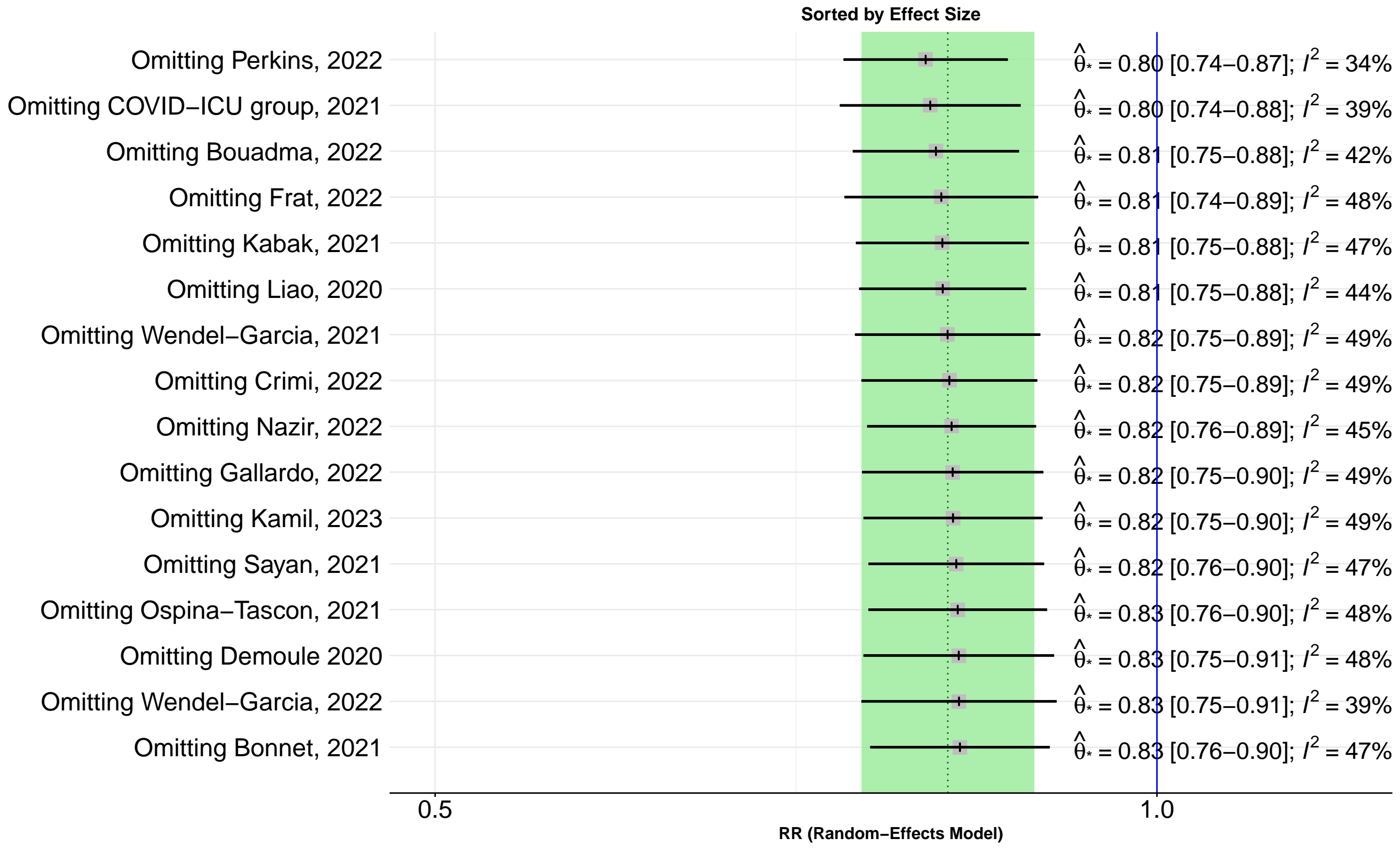

Supplement: Supplementary file 1 — Additional file 1: Figure S1. Risk of bias graph (ROB 2) for intubation outcome from randomized controlled trials. Figure S2. Funnel plot for intubation rate and assessment of small-study effects by Rücker’s limit meta-analysis method using Arcsine difference and Peters arcsine test. Figure S3. Funnel plot for mortality rate and assessment of small-study effects by Rücker’s limit meta-analysis method using arcsine difference and Peters arcsine test. Figure S4. Forest plot of intubation rate comparison between HFNC and COT from prospective and retrospective studies (random-effects meta-analysis by the Mantel–Haenszel method). COT, conventional oxygen therapy; HFNC, high-flow nasal cannula; M-H, Mantel–Haenszel. Figure S5. Sensitivity analysis of the risk of intubation through the leave-one-out strategy for the randomized controlled trials (fixed-effects meta-analysis by the Mantel–Haenszel method). COT, conventional oxygen therapy; HFNC, high-flow nasal cannula. Figure S6. Sensitivity analysis of the risk of intubation through the leave-one-out strategy for all studies (random-effects meta-analysis by the Mantel–Haenszel method). COT, conventional oxygen therapy; HFNC, high-flow nasal cannula. Figure S7. Forest plot of intubation rate comparison between HFNC and COT from randomized controlled trials according to the location of admission (random-effects meta-analysis by the Mantel–Haenszel method). COT, conventional oxygen therapy; HFNC, high-flow nasal cannula; ICU, intensive care unit; M-H, Mantel–Haenszel. Figure S8. Forest plot of mortality comparison between HFNC and COT from prospective and retrospective studies (random-effects meta-analysis by the Mantel–Haenszel method). COT, conventional oxygen therapy; HFNC, high-flow nasal cannula; M-H, Mantel–Haenszel. Figure S9. Forest plot of mortality rate comparison between HFNC and COT from randomized controlled trials according to the location of admission (fixed-effects meta-analysis by the Mantel–Haenszel method). [file 13613_2023_1208_MOESM1_ESM.zip › Supplementary/Supplementary figure S6-LeaveOneOut_AllStudies_Intubation.pdf]

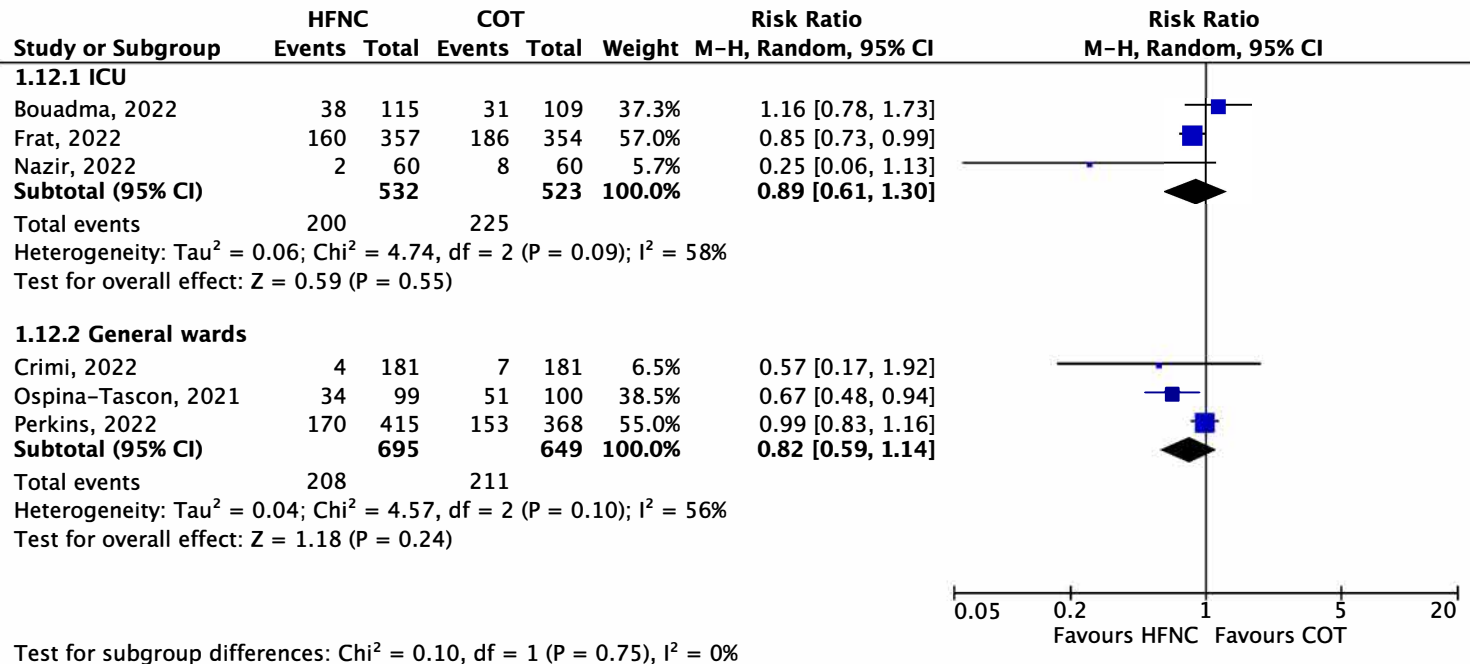

Supplement: Supplementary file 1 — Additional file 1: Figure S1. Risk of bias graph (ROB 2) for intubation outcome from randomized controlled trials. Figure S2. Funnel plot for intubation rate and assessment of small-study effects by Rücker’s limit meta-analysis method using Arcsine difference and Peters arcsine test. Figure S3. Funnel plot for mortality rate and assessment of small-study effects by Rücker’s limit meta-analysis method using arcsine difference and Peters arcsine test. Figure S4. Forest plot of intubation rate comparison between HFNC and COT from prospective and retrospective studies (random-effects meta-analysis by the Mantel–Haenszel method). COT, conventional oxygen therapy; HFNC, high-flow nasal cannula; M-H, Mantel–Haenszel. Figure S5. Sensitivity analysis of the risk of intubation through the leave-one-out strategy for the randomized controlled trials (fixed-effects meta-analysis by the Mantel–Haenszel method). COT, conventional oxygen therapy; HFNC, high-flow nasal cannula. Figure S6. Sensitivity analysis of the risk of intubation through the leave-one-out strategy for all studies (random-effects meta-analysis by the Mantel–Haenszel method). COT, conventional oxygen therapy; HFNC, high-flow nasal cannula. Figure S7. Forest plot of intubation rate comparison between HFNC and COT from randomized controlled trials according to the location of admission (random-effects meta-analysis by the Mantel–Haenszel method). COT, conventional oxygen therapy; HFNC, high-flow nasal cannula; ICU, intensive care unit; M-H, Mantel–Haenszel. Figure S8. Forest plot of mortality comparison between HFNC and COT from prospective and retrospective studies (random-effects meta-analysis by the Mantel–Haenszel method). COT, conventional oxygen therapy; HFNC, high-flow nasal cannula; M-H, Mantel–Haenszel. Figure S9. Forest plot of mortality rate comparison between HFNC and COT from randomized controlled trials according to the location of admission (fixed-effects meta-analysis by the Mantel–Haenszel method). [file 13613_2023_1208_MOESM1_ESM.zip › Supplementary/Supplementary figure S7_IOT_Subgroup_ICU_Wards.pdf]

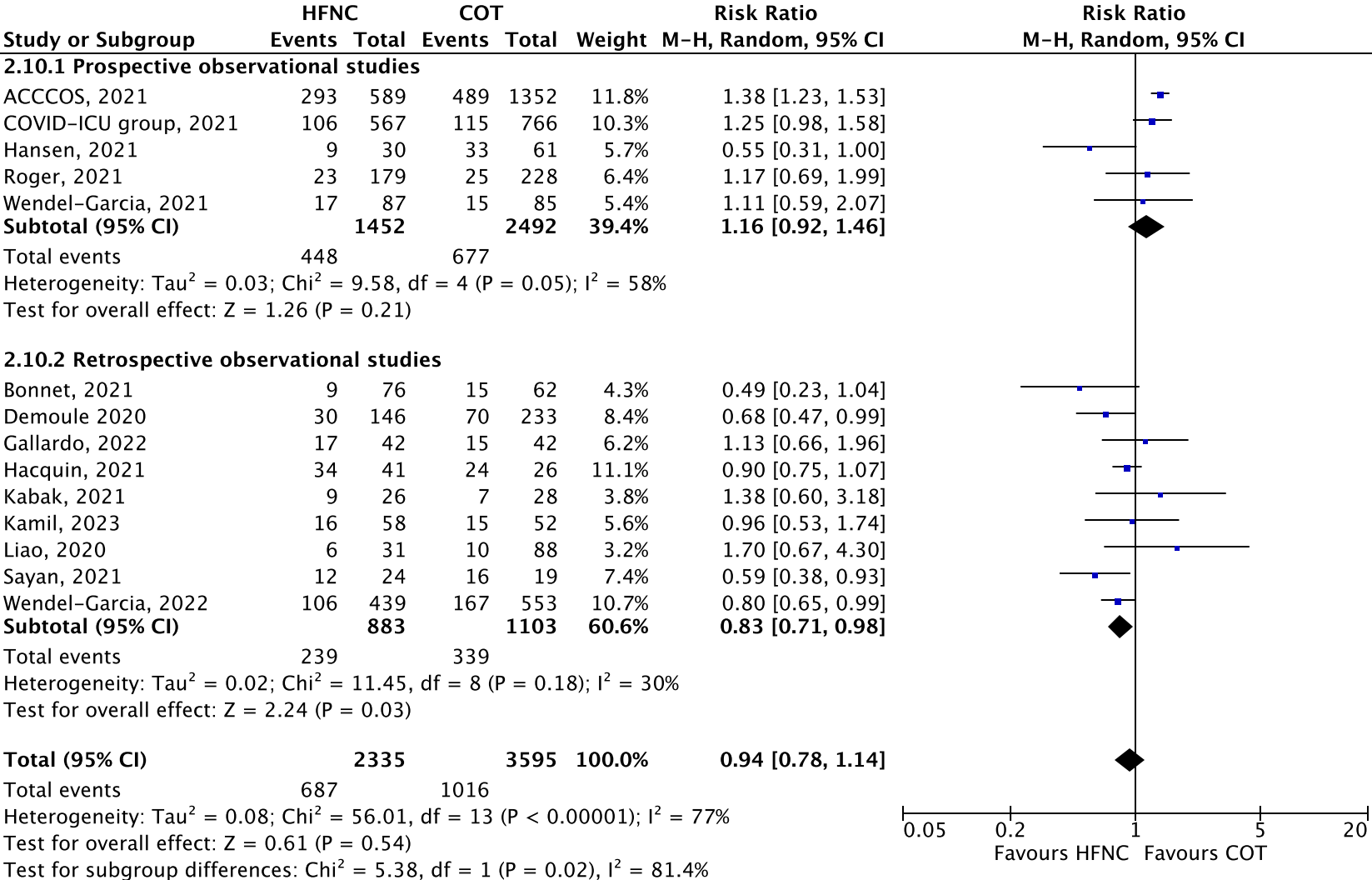

Supplement: Supplementary file 1 — Additional file 1: Figure S1. Risk of bias graph (ROB 2) for intubation outcome from randomized controlled trials. Figure S2. Funnel plot for intubation rate and assessment of small-study effects by Rücker’s limit meta-analysis method using Arcsine difference and Peters arcsine test. Figure S3. Funnel plot for mortality rate and assessment of small-study effects by Rücker’s limit meta-analysis method using arcsine difference and Peters arcsine test. Figure S4. Forest plot of intubation rate comparison between HFNC and COT from prospective and retrospective studies (random-effects meta-analysis by the Mantel–Haenszel method). COT, conventional oxygen therapy; HFNC, high-flow nasal cannula; M-H, Mantel–Haenszel. Figure S5. Sensitivity analysis of the risk of intubation through the leave-one-out strategy for the randomized controlled trials (fixed-effects meta-analysis by the Mantel–Haenszel method). COT, conventional oxygen therapy; HFNC, high-flow nasal cannula. Figure S6. Sensitivity analysis of the risk of intubation through the leave-one-out strategy for all studies (random-effects meta-analysis by the Mantel–Haenszel method). COT, conventional oxygen therapy; HFNC, high-flow nasal cannula. Figure S7. Forest plot of intubation rate comparison between HFNC and COT from randomized controlled trials according to the location of admission (random-effects meta-analysis by the Mantel–Haenszel method). COT, conventional oxygen therapy; HFNC, high-flow nasal cannula; ICU, intensive care unit; M-H, Mantel–Haenszel. Figure S8. Forest plot of mortality comparison between HFNC and COT from prospective and retrospective studies (random-effects meta-analysis by the Mantel–Haenszel method). COT, conventional oxygen therapy; HFNC, high-flow nasal cannula; M-H, Mantel–Haenszel. Figure S9. Forest plot of mortality rate comparison between HFNC and COT from randomized controlled trials according to the location of admission (fixed-effects meta-analysis by the Mantel–Haenszel method). [file 13613_2023_1208_MOESM1_ESM.zip › Supplementary/Supplementary figure S8_Mortality_Subgroup_Studies.pdf]

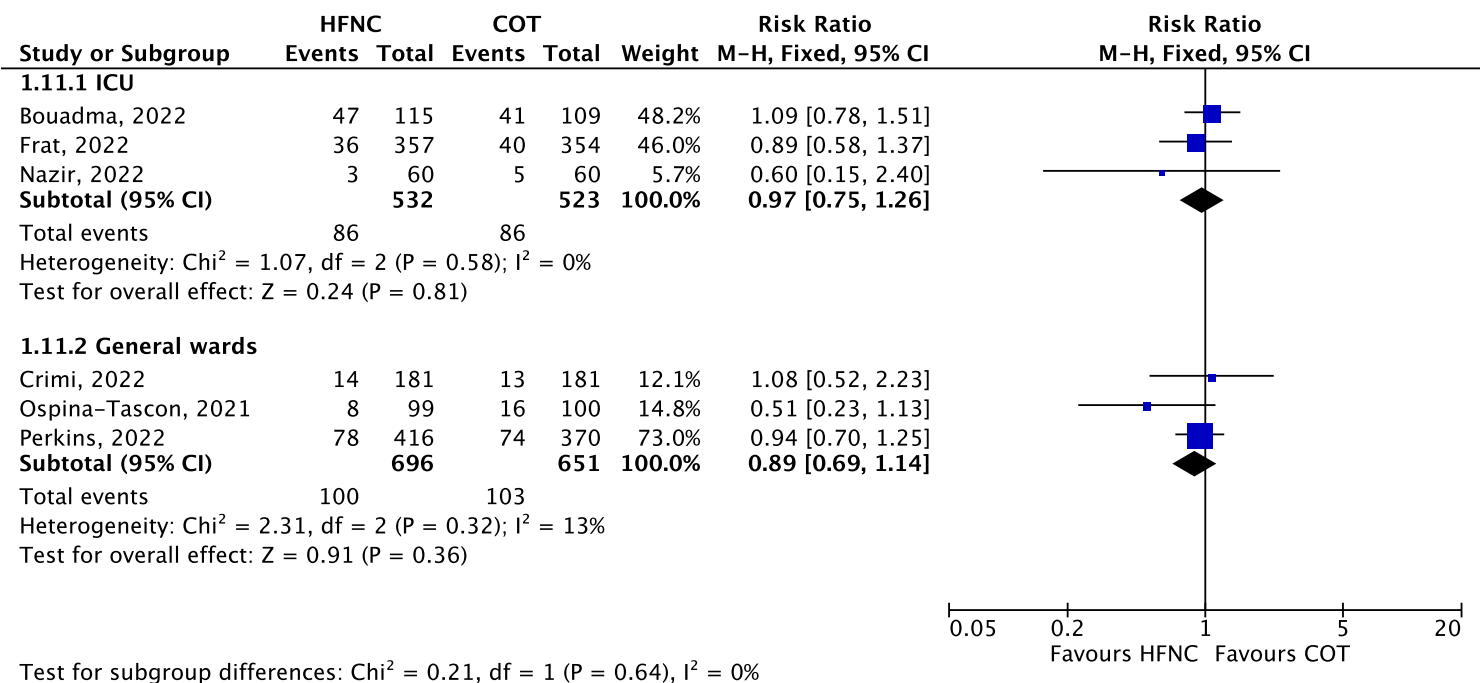

Supplement: Supplementary file 1 — Additional file 1: Figure S1. Risk of bias graph (ROB 2) for intubation outcome from randomized controlled trials. Figure S2. Funnel plot for intubation rate and assessment of small-study effects by Rücker’s limit meta-analysis method using Arcsine difference and Peters arcsine test. Figure S3. Funnel plot for mortality rate and assessment of small-study effects by Rücker’s limit meta-analysis method using arcsine difference and Peters arcsine test. Figure S4. Forest plot of intubation rate comparison between HFNC and COT from prospective and retrospective studies (random-effects meta-analysis by the Mantel–Haenszel method). COT, conventional oxygen therapy; HFNC, high-flow nasal cannula; M-H, Mantel–Haenszel. Figure S5. Sensitivity analysis of the risk of intubation through the leave-one-out strategy for the randomized controlled trials (fixed-effects meta-analysis by the Mantel–Haenszel method). COT, conventional oxygen therapy; HFNC, high-flow nasal cannula. Figure S6. Sensitivity analysis of the risk of intubation through the leave-one-out strategy for all studies (random-effects meta-analysis by the Mantel–Haenszel method). COT, conventional oxygen therapy; HFNC, high-flow nasal cannula. Figure S7. Forest plot of intubation rate comparison between HFNC and COT from randomized controlled trials according to the location of admission (random-effects meta-analysis by the Mantel–Haenszel method). COT, conventional oxygen therapy; HFNC, high-flow nasal cannula; ICU, intensive care unit; M-H, Mantel–Haenszel. Figure S8. Forest plot of mortality comparison between HFNC and COT from prospective and retrospective studies (random-effects meta-analysis by the Mantel–Haenszel method). COT, conventional oxygen therapy; HFNC, high-flow nasal cannula; M-H, Mantel–Haenszel. Figure S9. Forest plot of mortality rate comparison between HFNC and COT from randomized controlled trials according to the location of admission (fixed-effects meta-analysis by the Mantel–Haenszel method). [file 13613_2023_1208_MOESM1_ESM.zip › Supplementary/Supplementary figure S9_Mortality_Subgroup_ICU_Wards.pdf]
